# TDP-43 regulates cancer-associated microRNAs

**DOI:** 10.1007/s13238-017-0480-9

**Published:** 2017-09-26

**Authors:** Xiaowei Chen, Zhen Fan, Warren McGee, Mengmeng Chen, Ruirui Kong, Pushuai Wen, Tengfei Xiao, Xiaomin Chen, Jianghong Liu, Li Zhu, Runsheng Chen, Jane Y. Wu

**Affiliations:** 1 CAS Key Laboratory of RNA Biology, Institute of Biophysics, Chinese Academy of Sciences, Beijing, 100101 China; 2 Research Network of Computational Biology, RNCB, Beijing, 100101 China; 3 Core Facility for Protein Research, Institute of Biophysics, Chinese Academy of Sciences, Beijing, 100101 China; 4 State Key Laboratory of Brain and Cognitive Science, Institute of Biophysics, Chinese Academy of Sciences, Beijing, 100101 China; 5 Department of Neurology, Center for Genetic Medicine, Lurie Cancer Center, Northwestern University Feinberg School of Medicine, Chicago, IL 60611 USA; 6 Guangdong Geneway Decoding Bio-Tech Co. Ltd, Foshan, 528316 China

**Keywords:** TDP-43, miRNA, cancer, migration, prognosis

## Abstract

Aberrant regulation of miRNA genes contributes to pathogenesis of a wide range of human diseases, including cancer. The TAR DNA binding protein 43 (TDP-43), a RNA/DNA binding protein associated with neurodegeneration, is involved in miRNA biogenesis. Here, we systematically examined miRNAs regulated by TDP-43 using RNA-Seq coupled with an siRNA-mediated knockdown approach. TDP-43 knockdown affected the expression of a number of miRNAs. In addition, TDP-43 down-regulation led to alterations in the patterns of different isoforms of miRNAs (isomiRs) and miRNA arm selection, suggesting a previously unknown role of TDP-43 in miRNA processing. A number of TDP-43 associated miRNAs, and their candidate target genes, are associated with human cancers. Our data reveal highly complex roles of TDP-43 in regulating different miRNAs and their target genes. Our results suggest that TDP-43 may promote migration of lung cancer cells by regulating miR-423-3p. In contrast, TDP-43 increases miR-500a-3p expression and binds to the mature miR-500a-3p sequence. Reduced expression of miR-500a-3p is associated with poor survival of lung cancer patients, suggesting that TDP-43 may have a suppressive role in cancer by regulating miR-500a-3p. Cancer-associated genes *LIF* and *PAPPA* are possible targets of miR-500a-3p. Our work suggests that TDP-43-regulated miRNAs may play multifaceted roles in the pathogenesis of cancer.

**Electronic supplementary material:**

The online version of this article (doi:10.1007/s13238-017-0480-9) contains supplementary material, which is available to authorized users.

## Introduction

MicroRNAs (miRNAs) are small non-protein-coding RNAs (ncRNAs) with important regulatory function in biological and pathogenic processes by modulating mRNA decay or translational control (Ambros, 2004). Since their discovery two decades ago, miRNAs have been identified in nearly every eukaryotic organism examined (Ambros, 2011; Bartel, 2009). Extensive studies have begun to reveal the complex roles that miRNAs play in various diseases, including neurodegenerative disorders and cancer (Esquela-Kerscher and Slack, 2006; Gascon and Gao, 2014). Specifically, aberrant expression of miRNA is found in different types of cancer, and multiple miRNAs have been shown to contribute to cancer development and progression (Kong et al., 2012;Parpart and Wang, 2013).

Production of miRNAs is a highly regulated, multi-step process (Czech and Hannon, 2011). Briefly, the full-length primary transcript of a miRNA gene (pri-miRNA) forms a hairpin structure that is trimmed by the Drosha complex. The resulting pre-miRNA contains a 5p-arm, a 3p-arm, and a hairpin loop connecting them; this loop is removed by the Dicer complex to release a miRNA/miRNA* complex. One arm is then selected by RNA-induced silencing complexes (RISCs) as the mature miRNA. Furthermore, recent high-throughput sequencing data have shown that many mature miRNAs have a number of isoforms referred to as isomiRs, which may have different function (Cummins et al., 2006; Landgraf et al., 2007; Morin et al., 2008; Ruby et al., 2006). Thus, arm selection and isomiRs are important aspects in the formation of mature miRNAs.

The TAR DNA-binding protein 43 (TDP-43) contains two RNA recognition motifs (RRMs) and a carboxyl-terminal glycine-rich domain (Lee et al., 2012). In addition to transcriptional regulation, TDP-43 plays multiple roles in post-transcriptional gene regulation, including pre-mRNA splicing, mRNA transport and translation (Ayala et al., 2011; Baralle et al., 2013; Lagier-Tourenne et al., 2010; Ratti and Buratti, 2016). As a component of the Drosha and Dicer complexes, TDP-43 promotes miRNA biogenesis (Buratti et al., 2010; Freischmidt et al., 2013; Gregory et al., 2004; Kawahara and Mieda-Sato, 2012; Kocerha et al., 2011; Zhang et al., 2013). TDP-43 binds to UG repeat sequences in various RNAs (Kuo et al., 2009; Buratti et al., 2010), including the terminal loops of pre-miRNAs (Kawahara and Mieda-Sato, 2012). Long noncoding and coding RNA targets of TDP-43 in human and mouse have been reported (Tollervey et al., 2011; Polymenidou et al., 2011; Sephton et al., 2011). However, though there were early studies of microRNA targets of TDP-43 using microarrays, RNA-Seq has not been used to systematically examine the role of TDP-43 in microRNA regulation. In addition, though previous studies have documented association of TDP-43 expression with cancer, the underlying mechanisms of this association remain to be elucidated (Fang et al., 2011; Postel-Vinay et al., 2012; Teittinen et al., 2012;Campos-Melo et al., 2014; Park et al, 2013).

Here, we report a systematic search of miRNAs that are regulated by TDP-43 using a knockdown-coupled RNA sequencing approach. A number of microRNAs regulated by TDP-43 have been identified. In addition, TDP-43 down-regulation altered the isomiR patterns and arm selection of a subset of miRNAs. Biochemical experiments showed that TDP-43 directly binds the mature sequences of a subset of the TDP-43 regulated miRNAs, including miR-423-3p and miR-500a-3p. We identified several putative TDP-43-regulated miRNAs that are closely associated with cancers, especially lung cancer. A functional annotation pipeline designed for this study identified TDP-43-regulated miRNAs that may play a role in non-small cell lung cancer (NSCLC). Remarkably, miR-423-3p promoted migration of lung cancer cells *in vitro*. In contrast, miR-500a-3p was significantly associated with longer survival of lung cancer patients and targeted two cancer-associated genes, *LIF* (leukemia inhibitory factor) and *PAPPA* (pregnancy-associated plasma protein A, pappalysin 1). Taken together, our study has revealed complex gene networks that may be regulated by TDP-43 in human cancers and suggests that TDP-43 may modulate the expression of a subset of miRNAs associated with human cancers.

## RESULTS

### TDP-43 regulates the expression of a variety of microRNAs

**Figure 1:**
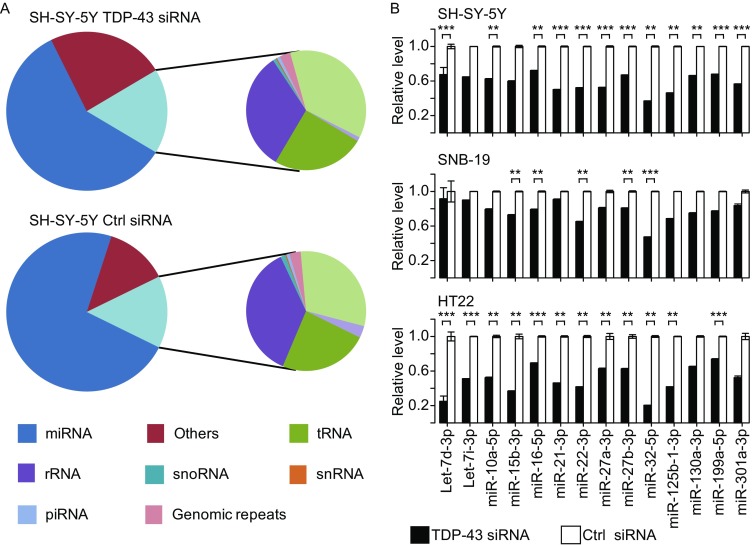
**RNA-seq analyses of TDP-43 regulated miRNAs**. Reads annotation and validation of small RNA sequencing in TDP-43 and control knockdown libraries. (A) Distribution of small RNA sequencing reads mapped to the human genome in SH-SY-5Y cells. Components of TDP-43 knockdown small RNA library (upper) and control library (bottom) are shown. (B) qRT-PCR of miRNAs that were differentially expressed following TDP-43 knockdown. The expression levels of mature miRNAs in SH-SY-5Y (upper panel), SNB-19 (middle), and HT22 (bottom) cells transfected with either TDP-43 siRNA or control (*n* = 3). Statistical analyses were performed using *t*-test. **P < 0.05; ***P < 0.01

To systematically search for miRNAs that are modulated by TDP-43, we performed RNA interference (siRNA) mediated TDP-43 knockdown in both human and mouse cells, specifically two human cell lines (neuroblastoma SH-SY5Y and glioblastoma SNB-19) and a mouse cell line with neuronal features, HT22 (see Fig. S1). Neuron-like cell lines were chosen because of the known role of TDP-43 in neurodegenerative diseases. Small RNAs were extracted from TDP-43 knockdown- and control cells and subjected to deep sequencing using an Illumina Genome Analyzer IIx system. From six sequenced libraries (one sample in each condition), we obtained 13.8–19.3 million reads for each sample of 86-nucleotides (nt) in length (Table S1). Most sequenced reads contained the adapter sequences at their 3′ ends. After trimming adapters and removing reads containing ambiguous bases, more than 95% of the total reads were useful and grouped into unique reads. The unique reads were analyzed and mapped to the human and mouse genomes (reference genomes hg19 and mm9, respectively). Approximately 55.6% of the total sequence reads could be mapped to the genomes without any sequence mismatch, and more than 27 million reads were usable for assessing RNA expression levels. The distributions of reads that could be aligned to tRNAs, rRNAs, snoRNAs, snRNAs, piRNAs, repeated sequences, and intron and exon sequences are shown in Table S1. The annotations of reads from TDP-43 knockdown and control SH-SY5Y cell lines are shown in Fig. 1A.

MicroRNAs whose expression was affected by TDP-43 knock-down were carefully analyzed. The reads counts were used to estimate the expression levels of miRNAs, as well as the expression levels of the isomiRs and 3′ single-nucleotide modified variants in the six libraries. Similar to a previous study (Morin et al., 2008), we used the most abundant variant to assess the expression level of the corresponding miRNA in the differential expression analysis.

Bayesian method (Audic and Claverie, 1997) was applied to identify the miRNAs whose expression may have changed. This method was originally designed to analyze differentially expressed genes through sequencing of their cDNA clones and has also been used to analyze small RNA sequencing data (Morin et al., 2008). In the SH-SY5Y cell line, TDP-43 knockdown resulted in 98 differentially expressed miRNAs (*P*-value < 0.001), of which 68 miRNAs were down-regulated. Of these 98 miRNAs, 2 miRNAs were up-regulated and 14 down-regulated after TDP-43 knockdown in the SNB-19 and HT22 cell lines. All of the differentially expressed miRNAs across the three cell lines are listed in Table S2. Of the 14 miRNAs down-regulated in all three cell lines from the RNA-Seq data, 11 were down-regulated in at least two cell lines, as validated by quantitative RT-PCR (Fig. 1B).

### TDP-43 knockdown alters isomiR composition

**Figure 2:**
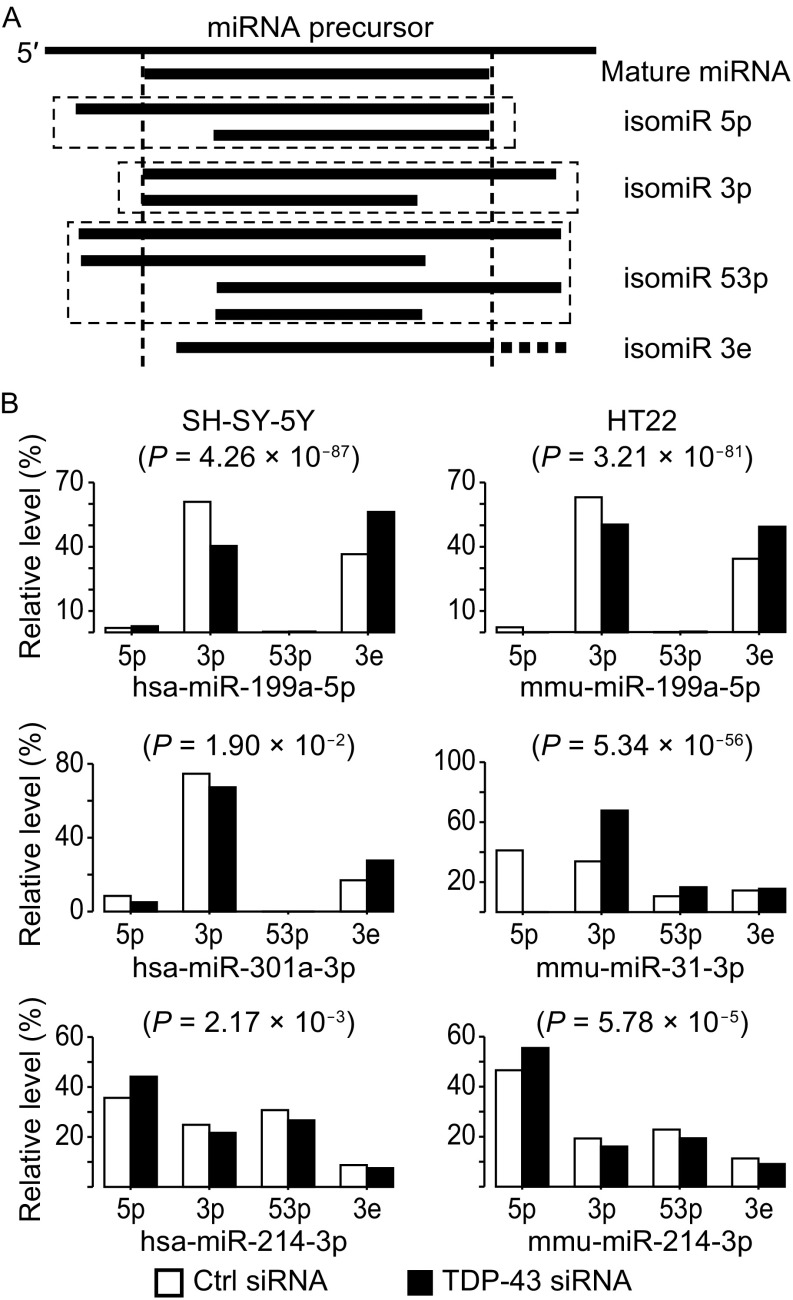
**TDP-43 knockdown leads to changes in miRNA isoform patterns**. (A) Definition of isomiR types. (B) IsomiR pattern alterations of hsa-miR-199a-5p, mmu-miR-199a-5p, hsa-miR-301a-3p, mmu-miR-31-3p, hsa-miR-214-3p, and mmu-miR-214-3p (Chi-square test)

The generation of isomiRs is probably caused by positional variation when Drosha and/or Dicer cleave precursor miRNAs (Morin et al., 2008). To compare the isomiR patterns between TDP-43 knockdown and control cell lines, we grouped the isomiRs into four types (Fig. 2A): isomiR-5p (5′ end addition or trimming), isomiR-3p (3′ end addition or trimming), isomiR-53p (simultaneous addition and/or trimming at both ends) and isomiR-3e (3′ end non-template single-nucleotide extensions).

Applying the chi-square test to the expression levels of the 4 types of isomiRs based on mature miRNA sequences from miRBase version 18 (Kozomara and Griffiths-Jones, 2011), we identified 9, 9 and 26 miRNAsin the SH-SY5Y, SNB19 and HT22 cells, respectively, that had significantly changed isomiR patterns after TDP-43 knockdown, in comparison to the control cells (Table S3; see MATERIALS AND METHODS). Changes in isomiR-3p were the most frequent among the altered isomiR patterns. Reduced expression levels of isomiR-3p after TDP-43 knockdown were frequently accompanied by increased expression of isomiR-3e, as observed for hsa-miR-199a-5p, hsa-miR-301a-3p, and mmu-miR-199a-5p (Fig. 2B). In contrast, the expression levels of isomiR-5p and isomiR-53p are less frequently altered after TDP-43 knockdown. Of note, isomiR-5p of miR-214-3p increased in both SH-SY-5Y and HT22 cells, and isomiR-53p of miR-31-3p increased in HT22 cells after TDP-43 knockdown (Fig. 2B).

### TDP-43 knockdown also alters miRNA arm selection

In order to estimate the possible effect of TDP-43 on strand selection from the miRNA duplex, we obtained reads counts of miRNAs processed from each arm of all precursors. Most precursors were processed into one dominant mature miRNA (i.e., from one arm), and the dominant arm was the same in all samples. Interestingly, we found several miRNAs where miRBase version 21 (Kozomara and Griffiths-Jones, 2014) annotates only one arm (either 5′ arm or 3′ arm) of their precursors could be processed into mature miRNA. However, we found that reads could map to both arms of their precursors, indicating a gap in its annotations (Table S4).

**Figure 3:**
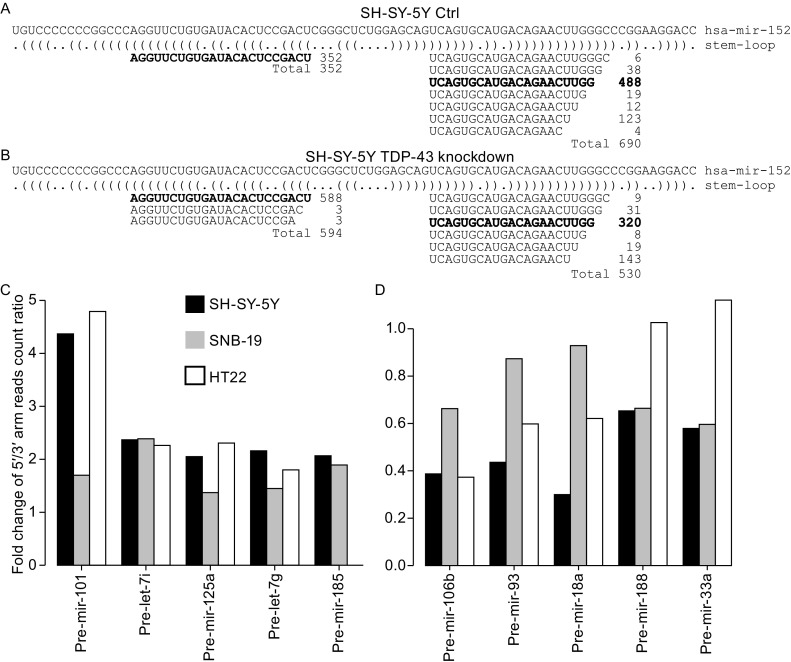
**Reads mapping to hsa-mir-152 and changes of 5′/3′ arm reads count ratio following TDP-43 knockdown**. Reads mapping to 5′ and 3′ arm of hsa-mir-152 in SH-SY-5Y control cells (A) and TDP-43 knockdown cells (B), mature miRNA sequences from both arms in bold. (C and D) Pre-miRNAs with more than 1.5 fold increase (C) or decrease (D) in 5′/3′ arm reads count ratio in at least two cell lines after TDP-43 knockdown

We calculated the 5′ arm/3′ arm reads count ratio for these miRNA precursors. For a certain fraction of the precursors, 5′ arm/3′ arm reads count ratio changed more than 1.5-fold after TDP-43 knockdown. For example, in the SH-SY5Ycell line, 12 precursors showed increased and 12 showed decreased 5′ arm/3′ arm reads count ratio after TDP-43 knockdown. Similar results were obtained for the other two cell lines (Tables S4 and S5). The most abundant miRNA with a changed arm ratio is miR-152, a miRNA known to be involved in various types of cancer (see discussion). In the SH-SY5Y control sample, the number of reads deriving from the 3′ arm of pre-mir-152 was nearly twice the number of reads deriving from the 5′ arm, whereas after TDP-43 knockdown the number of reads from the 5′ arm was about 12% higher than from the 3′ arm. Reads derived from the 5′ arm of pre-mir-152 after TDP-43 knockdown were of 3 different isoforms (Fig. 3A and 3B). The ratios of five pre-miRNAs (pre-let-7g, pre-let-7i, pre-mir-101, pre-mir-125a, and pre-mir-185) increased in at least two cell lines after TDP-43 knockdown, whereas the ratios of five other pre-miRNAs (pre-mir-106b, pre-mir-188, pre-mir-18a, pre-mir-33a, and pre-mir-93) decreased in at least two cell lines after TDP-43 knockdown (Fig. 3C and 3D). Together, these data clearly demonstrate that down-regulation of TDP-43 alters miRNA arm selection.

### Novel predicted miRNAs and miRNA arms

Among the more than 100 million reads from high-throughput sequencing of the 6 libraries, there was also a large amount of reads that mapped to un-annotated genomic regions. To identify novel miRNA candidates among the unknown reads, we first selected unique reads represented by more than 3 reads counts and mapped these to genomic regions with a perfect match. We applied the updated miRanalyzer webserver (Hackenberg et al., 2011) to identify novel miRNA candidates. By including only unique reads with more than 50 reads counts, we predicted 8 novel candidate miRNAs in human cell lines and 2 in the mouse cell line (Table S6). The flanking sequences of these novel miRNA candidates’ genome loci can be folded into stem-loop structures compatible with those of known pre-miRNAs. Most of these novel miRNA candidates were only detected after TDP-43 knockdown, suggesting that TDP-43 may inhibit the expression of these novel miRNA candidates. These novel miRNA candidates will be further investigated in future studies.

### TDP-43 interacts with the mature sequences of miR-423-3p and miR-500a-3p

**Figure 4:**
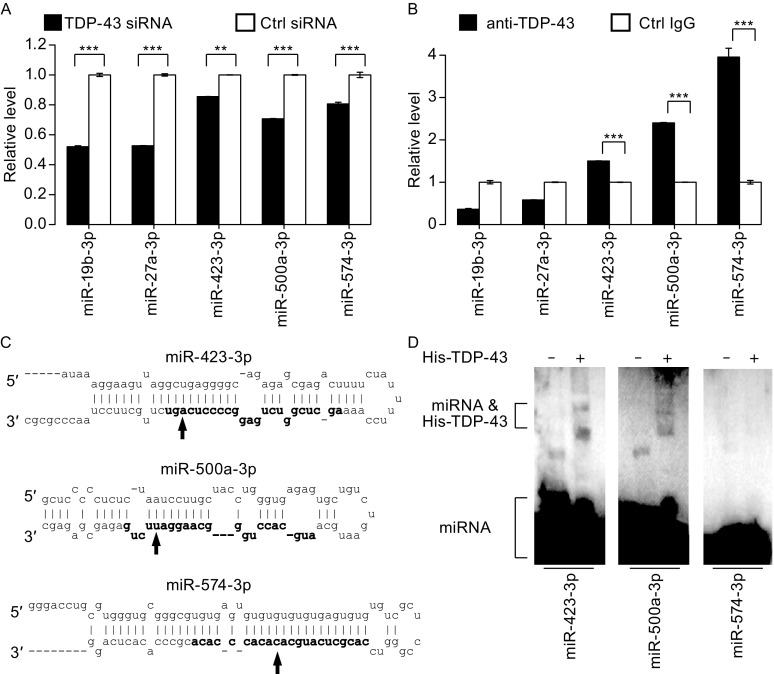
**Identification of TDP-43 binding miRNAs**. (A) Changes of expression levels of 5 miRNAs after TDP-43 knockdown in SH-SY-5Y cells. Relative miRNA levels were determined by qRT-PCR (*n* = 3; means ± SEM; **P < 0.05; ***P < 0.01). (B) Association between endogenous miRNAs and TDP-43. RNA Immunoprecipitation (RIP) enrichment was determined as miRNAs associated to TDP-43 IP relative to control IgG (*n* = 3; means ± SEM; **P <0.05; ***P <0.01). (C) TDP-43 binding sites in 3 different miRNAs. The secondary structures of the pre-miRNAs are shown, mature miRNAs sequences in bold. Predicted TDP-43 binding sites in the mature miRNAs are indicated by arrows. (D) Electrophoretic mobility shift assay (EMSA) with recombinant His-TDP-43 protein and biotin-labeled mature miRNA sequences. miR-423-3p (left), miR-500a-3p (middle), and miR-574-3p (right)

To further study miRNAs regulated by TDP-43, we examined which miRNAs interact with TDP-43. Cross-referencing raw data from a recent study using individual-nucleotide resolution cross-linking immunoprecipitation (iCLIP) approach (Tollervey et al., 2011), we found that TDP-43 might bind directly to the mature sequences of five miRNAs that were down-regulated after knockdown of TDP-43 in at least one of our cell lines. Because four of these (all but miR-27a-3p) were not included in the previous qRT-PCR (see Fig. 1B), we assayed the expression of miR-27a-3p and four other miRNAs in SH-SY-5Y cells by qRT-PCR, and found that the expression levels of all five miRNAs decreased significantly after knockdown of TDP-43 (Fig. 4A). To corroborate the interaction between TDP-43 and these miRNAs suggested by the raw iCLIP data, we assessed their interaction with an RNA immunoprecipitation (RIP) assay, which is similar to iCLIP, but without a UV cross-linking step (Peritz et al, 2006). Our RIP assay showed that three of the mature miRNAs (miR-423-3p, miR-500a-3p, and miR-574-3p) might bind to TDP-43 in SH-SY-5Y cells (Fig. 4B). Their potential binding sites, as indicated by the iCLIP data, are shown in Fig. 4C. Because RIP cannot unambiguously determine direct RNA-protein interactions (Ule et al, 2005; Moore and Silver, 2008), we further examined the direct interaction between TDP-43 and mature miRNAs in an electrophoretic mobility shift assay using purified TDP-43 protein (EMSA; Gangnon and Maxwell, 2011). Our data indicate that the TDP-43 protein binds miR-423-3p and miR-500a-3p, but not miR-574-3p (Fig. 4D). The data from all three methods (iCLIP, RIP, EMSA) corroborate that TDP-43 directly interacts with miR-423-3p and miR-500a-3p.

### A number of miRNAs affected by TDP-43 knockdown are involved in lung cancer

We identified several miRNAs whose expression levels, arm selection or isomiR patterns were regulated by TDP-43 from small RNA sequencing analyses. Interestingly, previous studies reported that at least 64 putative TDP-43-regulated miRNAs are associated with cancers as either oncogenic factors or tumor suppressors (Table S7), suggesting an association between TDP-43 and cancer via miRNAs. Extracting annotations of these miRNAs from the miR2Disease webserver (Jiang et al., 2009), 39 of them have previously been associated with lung cancer (Table S8), a leading cause of cancer-related deaths. The role of TDP-43 associated miRNAs in lung cancer remains unclear. Therefore, we decided to focus our efforts on the possible role of TDP-43 in lung cancer.

In order to examine how TDP-43 might be involved in lung cancer via the miRNAs that it regulates, we designed an analysis pipeline that combined ProMISe (probabilistic miRNA-mRNA interaction signature) (Li et al., 2014), DESeq2 (Love et al., 2014), Fatiscan (Al-Shahrour et al., 2007a), and FatiGO (Al-Shahrour et al., 2007b). See Figure S2 for a diagrammatic illustration of the analysis pipeline. There are three inputs: the preprocessed counts provided by the TCGA data for miRNAs and mRNAs, as well as initial preprocessed predictions from miRanda (Betel et al., 2010). The pipeline includes 5 steps: 1) to use DESeq2 to determine which miRNAs and transcripts are differentially expressed; 2) to generate predicted miRNA-mRNA interactions for each sample using the initial miRanda predictions and ProMISe; 3) to extract the interactions found in all samples, then use Fatiscan to identify whether the set of targets for a miRNA are enriched for differentially expressed targets; 4) to filter the resulting list for miRNAs that are differentially expressed by DESeq2 and that have evidence of regulation by TDP-43; 5) finally, to identify which processes the miRNAs are regulating by using FatiGO to look for overrepresented gene ontology and pathway terms. In this study, we focused on the two datasets studying NSCLC, including lung squamous cell carcinoma (LUSC) and lung adenocarcinoma (LUAD), because NSCLC accounts for the majority of lung cancer cases. We included samples that had paired miRNA-mRNA expression profiles available from The Cancer Genome Atlas as of July 2014 (Collins and Barker, 2007).

First, the miRNAs were tested for differential expression in LUAD or LUSC versus control samples using DESeq2. Out of the 1,100 human miRNAs in the miRBase v21 database (Kozomara and Griffiths-Jones, 2014) 417 and 563 miRNAs showed differential expression in LUAD and LUSC samples, respectively, versus control samples. MiRNAs putatively regulated by TDP-43 were over-represented in these groups (hypergeometric test *P*-value for LUAD (57/417 vs. 83/1033) and LUSC (61/563 vs. 83/1037): 5.24 × 10^−8^ and 1.48 × 10^−4^, respectively). Because TDP-43 may not regulate all of these miRNAs, we examined the correlation between each miRNA and TDP-43 expression in lung cancer samples. We performed Pearson correlation, and after correcting for multiple hypothesis testing, identified 408 and 467 miRNAs significantly correlated with TDP-43 in LUAD and LUSC samples, respectively (FDR < 0.1). MiRNAs putatively regulated by TDP-43 had a trend for overrepresentation in these groups as well (hypergeometric test *P*-value for LUAD (39/408 vs. 83/1033) and LUSC (45/467 vs. 83/1037): 0.091 and 0.059, respectively).

To identify which miRNAs showed enrichment for differentially expressed targets, we used the combination of ProMISe and Fatiscan. From the ProMISe step, out of the 1033 and 1037 miRNAs that were expressed in LUAD and LUSC samples respectively, there were 213 and 274 miRNAs that had at least 5 predicted targets in each LUAD or LUSC sample, respectively; miRNAs regulated by TDP-43 were also over-represented in these groups (hypergeometric test *P*-value for LUAD (67/213 vs. 83/1033) and LUSC (74/274 vs. 83/1037): 3.36 × 10^−35^ and 1.89 × 10^−36^, respectively). A ranked list of the transcripts (using *P*-values of log fold change and log differential expression from DESeq2) was submitted along with the predicted miRNA-mRNA interactions from ProMISe as custom annotations to Fatiscan (part of the Babelomics v4 suite; Medina et al., 2010). This method uses a threshold-independent heuristic to identify whether an annotation term is over-represented in the bottom or top of a ranked list; and in this case, we were searching for miRNAs that had an enrichment for either up-regulated or down-regulated target transcripts.

In order to identify miRNAs that were both differentially expressed and had enrichment for differentially expressed targets, we combined the Fatiscan results with earlier DESeq2 results for the miRNAs to get a joint *P*-value. We applied three criteria to filter the list of miRNAs to the most relevant one: (A) the miRNA had to have a DESeq2 differential expression FDR < 0.1; (B) the targets of the miRNA had to be changing in the opposite direction (if the miRNA is up-regulated, the targets must be down-regulated, and vice versa); (C) the miRNA expression profile had to have a statistically significant correlation with TDP-43 expression profile (FDR < 0.1). The results of this step for LUAD and LUSC are shown in Tables S9 and S10, respectively.

To determine what biological processes were related to the identified targets, we extracted the unique transcripts from the previous step and submitted them to FatiGO, also part of the Babelomics v4 suite; this tool performs an overrepresentation analysis for gene ontology and pathway terms. Among the down-regulated transcripts, the most significant hits included integrin cell surface interactions and negative regulation of cell proliferation; among the up-regulated transcripts, the most significant hits included nucleotide synthesis, cell cycle checkpoints, and RNA processing. This suggests that TDP-43-regulated miRNAs may play a role in promoting carcinogenesis and metastasis. The full list of hits can be found in Table S11.

From this analysis pipeline, we defined “predicted causal interactions” as those miRNA-mRNA interactions between putative TDP-43-regulated miRNAs and target mRNA transcripts with annotations in the processes discovered by FatiGO. See Figure S3A and S3B for the representative network graph of up-regulated miRNAs and down-regulated transcripts in LUAD (one network of 7 up-regulated miRNAs, 50 down-regulated transcripts, and 13 processes; another network of 4 down-regulated miRNAs, 62 transcripts, and 17 processes), and Fig. S3C and S3D for the LUSC network, which was much larger. See Table S12 for the full node and edge lists.

In summary, our analysis pipeline identified a number of putative TDP-43-regulated miRNAs which target several transcripts that have roles in cancer biology, including the two TDP-43 interacting miRNAs identified in this study, miR-423-3p and miR-500a-3p. These were experimentally examined further for their roles in lung cancer.

### TDP-43 associated miR-423-3p promotes lung cancer cell migration

From our analysis pipeline, miR-423-3p was one miRNA that met all of our criteria in LUSC samples: it was differentially expressed, significantly correlated with TDP-43, and had targets with statistically overrepresented pathway annotations. Of the four targets that were hits, three (CRK, LCP2, and ITGA9) were related to the reactome pathway “Integrin Cell Surface Interactions”; even when including the rest of the differentially expressed targets of miR-423-3p, this was the only significant pathway identified by FatiGO. Thus, we hypothesized that TDP-43 might influence lung cancer cell migration via miR-423-3p.

**Figure 5:**
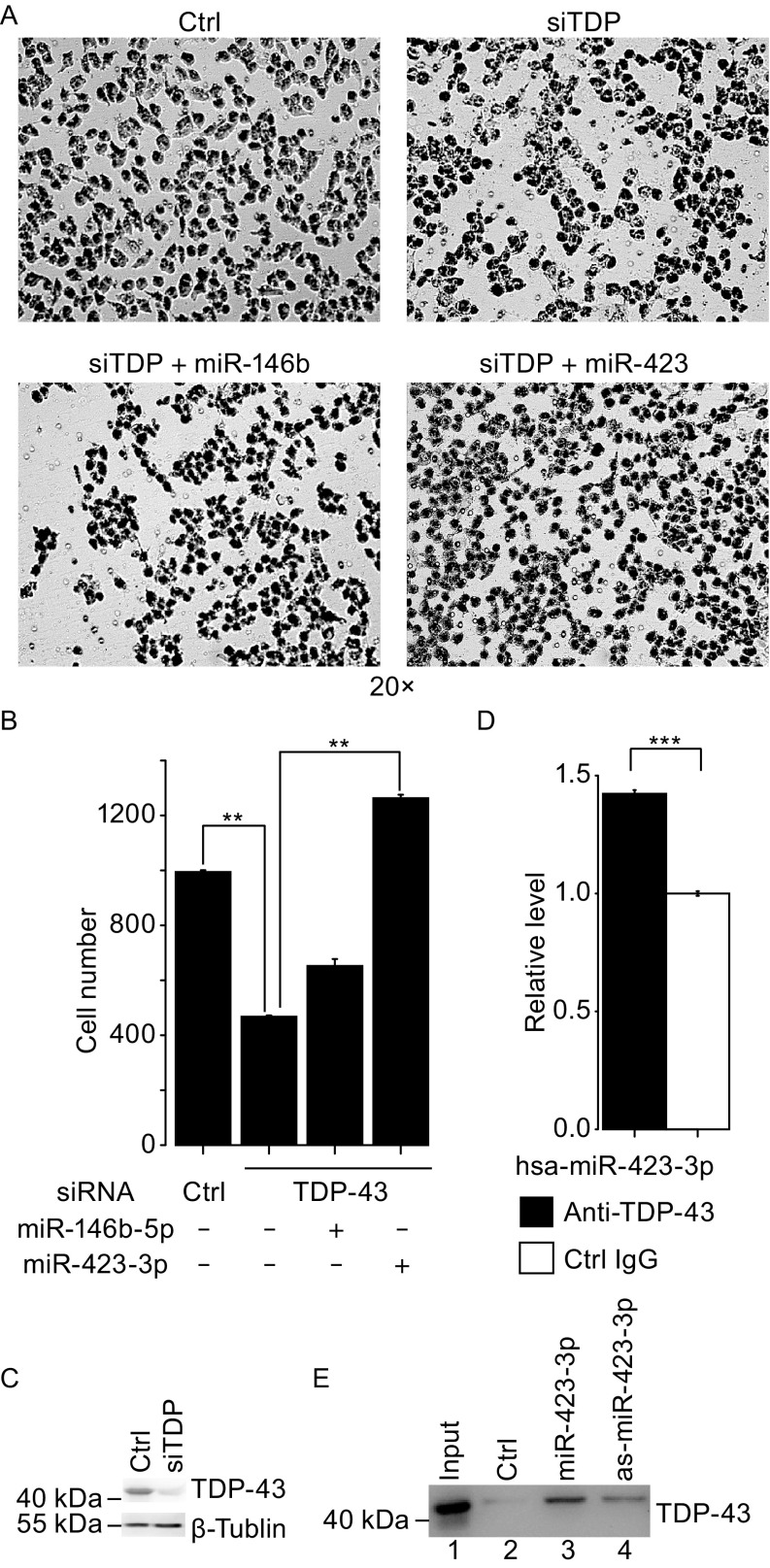
**Effect of TDP-43 regulated miRNAs on lung cancer cell migration as detected by trans-well migration assay**. (A) H1299 cell migration after transfection with either control siRNAs or TDP-43 siRNAs alone (upper panel) or in combination with has-miR-146b-5p (bottom left) or has-miR-423-3p (bottom right). (B) Quantification of cells migrated cross the membrane. (C) Western blotting showing the reduction of TDP-43 expression H1299 cells treated with siTDP-43 as compared with the control siRNA (Ctrl). (D) RIP-coupled qRT-PCR assay of interaction between TDP-43 and has-miR-423 in H1299 cells. Enrichment was determined as miR-423 associated to TDP-43 IP relative to control IgG. (E) RNA pull-down assays of the interaction between has-miR-423 and TDP-43 (combined with qRT-PCR) in H1299 cells. Lane 1, ~3% input; Lane 2, negative control; Lane 3 and 4, biotinylated has-miR-423-3p and antisense-hsa-miR-423-3p, respectively. (*n* = 3; means ± SEM; **P < 0.05; ***P < 0.01)

To test this hypothesis, we performed TDP-43 knockdown on H1299 lung cancer cells and measured cell migration using the transwell migration assay. Our data showed a significant reduction in cell migration by TDP-43 knockdown (Fig. 5A–C). In order to address whether this inhibition of cell migration was caused by TDP-43 regulated miRNAs, we co-transfected H1299 cells with TDP-43 siRNAs and each of six miRNAs: miR-423-3p and five others that were previously identified to be related to lung cancer (see Table S8). After co-transfection with miR-423-3p, the cell migration increased significantly (*P*-value < 0.05) as compared with cells transfected with TDP-43 siRNAs alone (Fig. 5B). Co-transfection with other miRNAs did not rescue the reduction in cell migration caused by TDP-43 knockdown (Fig. 5A and 5B). Similar to results from SH-SY5Y cells, examination of the interaction between miR-423-3p and TDP-43 using RNA immunoprecipitation (RIP) and RNA pull-down assay showed that miR-423-3p interacts with TDP-43 in H1299 lung cancer cells (Fig. 5D and 5E). Thus, these results suggest that TDP-43 may promote lung cancer cell migration through regulation of miR-423-3p, corroborating the prediction from the functional annotation pipeline that TDP-43 is a tumor-promoting factor.

### TDP-43 regulated miRNAs, including miR-500a-3p, may serve as prognostic markers of cancers

Among the TDP-43 affected miRNAs that we identified, the expression levels of 17 miRNAs have previously been associated with NSCLC patient survival. Similarly, in glioblastoma multiforme (GBM), the expression levels of 18 TDP-43 regulated miRNAs might have prognostic value for GBM (Table S13).

In order to test the association between TDP-43 regulated miRNAs and patient survival in cancer using samples independent from these previous studies, we collected miRNA expression profiles of 134 lung squamous cell carcinoma (LUSC), 191 lung adenocarcinoma (LUAD) and 487 GBM, and gene expression profiles of 133 LUSC, 231 LUAD and 538 GBM from The Cancer Genome of Atlas (TCGA), together with information on the patient survival. We found that 4 of the 17 TDP-43 regulated miRNAs in NSCLC were also significantly associated with patient survival in the independent cohort (let-7b-5p, miR-25-3p, miR-31-5p, and miR-93-5p), and that 5 of the 18 miRNAs were confirmed to be significantly associated with GBM patient survival in this independent cohort (miR-148a-3p, miR-17-5p, miR-20a-5p, miR-221-3p, and miR-31-5p) (Table S13). Low expression of hsa-let-7b-5p and high expression of hsa-miR-31-5p were associated with poor survival in lung cancer, which is consistent with previous reports (Fig. S4; Tan et al., 2011; Yanaihara et al., 2006), and hsa-let-7b was identified as a hit from our pipeline. Low expression of miR-17-5p and miR-20a-5p and high expression of miR-148a-3p, miR-221-3p, and miR-31-5p were associated with poor prognostic outcome in GBM independent cohort (Fig. S4). The associations of these 5 miRNAs with patient survival have all been reported previously (Delfino et al., 2011; Srinivasan et al., 2011).

**Figure 6:**
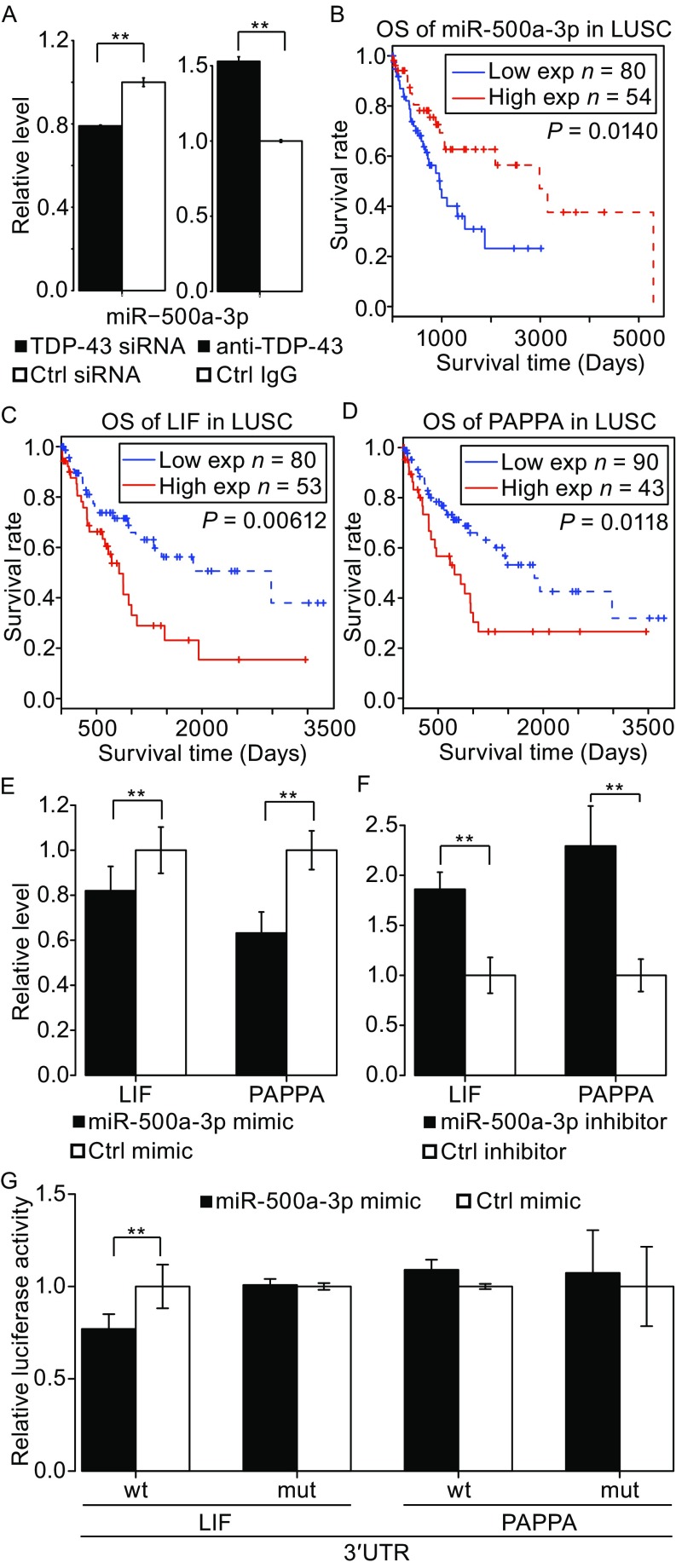
**Overall survival analyses of miR-500a-3p and its target genes in lung cancer**. (A) Effect of TDP-43 depletion on miR-500a-3p expression level in H1299 cells as determined by qRT-PCR (left panel). Interaction between TDP-43 and miR-500a-3p as assayed by RIP-coupled qRT-PCR (right panel). (*n* = 3; means ± SEM; **P < 0.05). (B) Overall survival analysis for miR-500a-3p in LUSC. (C and D) Overall survival analysis for the miR-500a-3p target genes LIF (C) and PAPPA (D) in LUSC. *P*-values from log-rank test. (E and F) MiR-500a-3p represses endogenous expression of *LIF* and *PAPPA* in H1299 cells. Over expression of miR-500a-3p represses *LIF* and *PAPPA* expression relative to control mimic (E). After transfection of a miR-500a-3p inhibitor, expressions of *LIF* and *PAPPA* are de-repressed compared to control inhibitor (F). (*n* = 3; means ± SEM; **P < 0.05). (G) The wild-type or mutated 3′UTR reporter constructs of LIF and PAPPA were transfected with control mimics or miR-500a-3p mimics into H1299 cells. Luciferase activities were measured 24 h after transfection. (*n* = 3; means ± SEM; **P < 0.05)

One of the miRNAs associated with NSCLC survival is miR-500a-3p. From our analysis pipeline, we identified miR-500a-3p to be significantly correlated with TDP-43. To examine the potential role of miR-500a-3p in lung cancer, we examined miR-500a-3p and its predicted target genes in H1299 cells. TDP-43 could bind to miR-500a-3p and regulate its expression level in H1299 cells (Fig. 6A, right panel and left panel respectively). The survival analysis of 134 LUSC samples indicates that higher expression level of miR-500a-3p is correlated with longer patient survival (Fig. 6B), and suggests that miR-500a-3p may be a protective factor in lung cancer patients. Potential target genes of miR-500a-3p were predicted using updated calculated predictions from three methods (see MATERIALS AND METHODS). Among the miR-500a-3p target genes, lower expression levels of 29 predicted target genes (of which 8 that were predicted by at least two methods) were associated with better survival of lung cancer patients (Table S14). In order to determine whether some of these predicted genes are targets for miR-500a-3p, we first overexpressed miR-500a-3p and examined the expression levels of predicted target genes. *LIF* and *PAPPA*, whose expression levels were associated with worse survival of lung cancer patients (Fig. 6C and 6D), were down-regulated in lung cancer H1299 cells after transfection of miR-500a-3p compared to control miRNA mimetic by qRT-PCR (Fig. 6E). Consistently, after knockdown of miR-500a-3p, *LIF* and *PAPPA* mRNA levels were up-regulated in H1299 cells (Fig. 6F). To determine whether miR-500a-3p interacts with the 3′UTR regions of *LIF* and *PAPPA* mRNAs, we co-transfected miR-500a-3p mimetic or control with luciferase reporter constructs for *LIF* or *PAPPA* containing the respective wild-type 3′UTR or the mutant 3′UTR containing mutated binding site for miR-500a-3p into H1299 cells. Mutating the miR-500a-3p binding site in the *LIF* 3′UTR reversed effect of miR-500a-3p (Figs. 6G and S5). Together these results suggest that *LIF* and *PAPPA* may be biological targets of miR-500a-3p in lung cancer cells.

## Discussion

MiRNAs are recognized as critical links in cellular pathways and carcinogenesis, regulating expression of genes in a wide range of pathways critical for cancer development and progression. MiRNAs regulate the expression of their target genes at the transcriptional and translational levels. They can also modulate the production or turnover of mRNAs. Dysregulation of miRNAs is associated with a variety of human diseases, such as cancers and neurological disorders (Calin and Croce, 2006; Maciotta et al., 2013; Nelson et al., 2008;Abu-Elneel et al, 2008;Jonas and Izaurralde 2015; Bracken et al, 2016;Adams et al, 2017).

Although significant efforts have been made in studying the role of TDP-43 in neurodegenerative diseases, its biological function is not fully understood. TDP-43 has been shown to participate in various aspects of RNA metabolism including transcription, pre-mRNA splicing, RNA transport and translational regulation (Ayala et al., 2011; Baralle et al., 2013; Lagier-Tourenne et al., 2010; Ratti and Buratti, 2016). RNA targets of TDP-43 have been studied by CLIP-Seq, showing that TDP-43 not only binds to mRNAs, long ncRNAs, snoRNAs and rRNAs, but also miRNAs (Buratti et al., 2013; Polymenidou et al., 2011; Tollervey et al., 2011; Xiao et al., 2011; reviewed in Ratti and Buratti 2016). TDP-43 is a known component of two important miRNA processing complexes (Drosha and Dicer) and associates with several auxiliary factors (Kawahara and Mieda-Sato, 2012a). Therefore, TDP-43 is critically involved in miRNA biogenesis.

Relationships between TDP-43 and cancer have been reported previously (Fang et al., 2011; Postel-Vinay et al., 2012; Teittinen et al., 2012; Park et al., 2013). Broadly speaking, TDP-43 may be involved in carcinogenesis through participating in the processes of miRNA production and turnover. In a recent example (Park et al., 2013), TDP-43 was found to inhibit the miR-520 family in hepatocellular carcinoma, which in turn disinhibited the platelet isoform of phosphofructose kinase (PFKP). To take another example, it was reported that in *Drosophila*, TDP-43 bound to pri-miR-9a to maintain its stability and the expression level of the mature sequence, and that TDP-43 conferred robustness to neuronal specification through miR-9a (Li et al., 2012b). Consistent with these results, our data show that both miR-9-5p and miR-9-3p were significantly down-regulated after TDP-43 knockdown (Table S2). Because hsa-miR-9-5p has been shown to contribute to breast cancer pathogenesis (Table S7; Ma et al., 2010), our data suggest that TDP-43 might play a role in promoting breast cancer through its interaction with miR-9. Finally, another study showed TDP-43 binds to both pre-let-7b and mature let-7b-5p (Buratti et al 2010); let-7b-5p was one of the hits in our computational pipeline and one miRNA that had prognostic value in LUAD patients.

Our study has systematically identified a subset of TDP-43-regulated miRNAs in the small RNA transcriptome by high-throughput sequencing. Our high-throughput sequencing data showed that the expression levels of a substantial number of miRNAs were altered in at least one of the three cell lines after TDP-43 knockdown. Many of the miRNAs affected by TDP-43 knockdown were down-regulated, but only 13 of the affected miRNAs have previously been reported to be affected by TDP-43 expression level changes (Buratti et al., 2010; Kawahara and Mieda-Sato, 2012,Li et al., 2012b). This lack of overlap may be explained by the published data being based on microarray-based studies. Compared with microarray, high-throughput sequencing may be a better choice in identifying differentially expressed miRNAs at low expression levels (Malone and Oliver, 2011), as miRNA expression profiles obtained from microarray may have higher false positive rate due to experimental bias and cross-hybridization (Hurd and Nelson, 2009). Among the 14 confidently predicted down-regulated miRNAs, the down-regulation was confirmed by qRT-PCR for more than 85% of the miRNAs. Because of the diverse spectrum of the miRNAs whose expression may be regulated by TDP-43, we chose to focus on those miRNAs closely associated with cancer in this study and will continue to perform in-depth analyses on other TDP-43-regulated miRNAs in a separate study.

IsomiRs are natural variants expressed from the same miRNA locus (Lee et al., 2010), and have been detected in a variety of species by high-throughput sequencing (Fernandez-Valverde et al., 2010; Humphreys et al., 2012; Liu et al., 2011; Luciano, 2004). Some specific isomiRs have also been associated with diseases, including cancer (e.g., Guo et al., 2011; Li et al., 2012a; Marti et al., 2010). The binding properties of isomiRs to their mRNA targets may vary (Baccarini et al., 2011). Marked differences in isomiR distributions were seen after TDP-43 knockdown. The expression levels of most miRNAs with altered isomiRs patterns were also significantly changed by TDP-43 knockdown, indicating a role of TDP-43 in miRNA editing, modification and/or turnover. These data suggest that TDP-43 may play a role in cancer pathogenesis by altering isomiR patterns.

The pre-miRNA contains three parts: the 5′ arm, the 3′ arm, and the terminal loop. Mature miRNAs are derived from either the 5′ arm or the 3′ arm, with unequal probabilities. A common view is that the arm preference is determined by the hydrogen-bonding-based selection rule (Khvorova et al., 2003; Schwarz et al., 2003). However, this view has been challenged by some recent studies (e.g., Griffiths-Jones et al., 2011). MiRNAs from the two opposite arms differ in potential mRNA targets and functions. For example, arm selection differences have been observed in gastric cancer (Li et al., 2012a). Our data show that the arm preference is altered by reduction of TDP-43 expression. In SH-SY-5Y cells, the favored arm of pre-mir-152 switched from the 3′ arm to the 5′ arm following TDP-43 knockdown. MiR-152 has been reported to be associated with different types of cancer, such as hepatocellular carcinoma (Huang et al., 2010), endometrial cancer (Tsuruta et al., 2011), ovarian cancer (Woo et al., 2012; Zhou et al., 2012), and gastrointestinal cancer (Chen et al., 2010). In addition, mir-152-3p was identified as a significant hit in LUSC samples from our computational analyses. TDP-43 may therefore act as a regulator of miR-152 processing in these cancers.

Among the TDP-43-regulated miRNAs with previously established connections with cancer, lung cancer was identified as the one with the largest number of associated miRNAs (38 from Table S7; 39 from Table S8). We set out to design a pipeline that would predict the processes that TDP-43 would affect their predicted target genes via its regulated miRNAs. There are some limitations to the approach taken: (1) if a target interaction was missing from even one sample, it was excluded from the set of targets analyzed for a miRNA; this made the analysis easier to interpret, but it excluded miRNA-mRNA interactions that are likely important for a subset of lung cancer samples; (2) only the most differentially expressed genes from the Fatiscan results were included for the FatiGO analysis; thus, any subtle but biologically important signals in the data were ignored; (3) we did not examine whether there were miRNA-mRNA interactions that were positively correlated, which can indicate miRNAs either enhancing mRNA expression (Orang et al., 2014) or acting in a “tuning” or noise-buffering capacity (Bartel, 2009; Noorbakhsh et al., 2013; Osella et al., 2011); (4) we cannot exclude the possibility of other miRNAs or other genes being the true cause of the changes we observe in the target gene. Despite these limitations, this pipeline provides a clear set of hypotheses for future work to validate.

The resultant predicted causal interaction network provides a complex picture of the predicted impact of TDP-43 on the pathogenesis of lung cancer. One aspect that complicates analysis has to do with the opposing roles of alternative isoforms. Several of the genes have only one transcript predicted to be a target, and this leads to a context-specific effect on cancer pathogenesis. For example, our pipeline predicted miR-423-3p to have four important targets in LUSC. Prima facie, the results seem mixed because two genes were shown to inhibit cell migration (LCP2 (Baker et al., 2009) and ADRB2 (Yu et al., 2007)), one showed mixed results with respect to cell migration (ITGA9; Mostovich et al., 2011), and another was reported to promote cell migration (CRK (Sriram and Birge, 2010)). However, when one looks more closely at the transcript level, the CRK transcript that is targeted is CrkIII, the shortest isoform with a predicted structure that has a truncated SH3 domain (Sriram and Birge, 2010). Thus, it is reasonable to propose that, given the established role of miR-423-3p in promoting cell migration, CrkIII may act as a competitive inhibitor of Crk signaling and that inhibition by miR-423-3p leads to restoration of Crk signaling that promotes cell migration. Future work is necessary to test this hypothesis and tease out the other complexities of transcript-specific miRNA targeting.

Another complicating aspect was how the miRNAs that were identified as hits in our pipeline have mixed roles in tumorigenesis. Of the 28 miRNAs reported as hits in either LUSC or LUAD samples, 22 have previous literature exploring their roles in cancer (12 miRNAs are indicated as suppressors and 10 are reported as oncomiRs; see Tables S7 and S8); there was a trend toward tumor suppressor TDP-43-regulated miRNAs being down-regulated (hypergeometric test *P*-value for LUAD (4/4 vs. 40/85) and LUSC (7/9 vs. 40/85) respectively: 0.045 and 0.054), which suggested that TDP-43 is a tumor promoter. However, this trend was not seen with oncomiRs overrepresented among up-regulated hits (*P*-values for LUAD and LUSC were 0.614 and 0.177 respectively).

Our data presented here suggest that TDP-43 may promote miRNA biogenesis and interact with miRNAs to regulate their function in cancers in a highly complex manner. On one hand, TDP-43 may have a role in promoting cancer development by regulating miR-423-3p. Increased expression of miR-423-3p has been reported in lung cancer patient samples (Crawford et al., 2009). MiR-423-3p has been shown to promote cell growth in human hepatocellular carcinoma cell lines (Lin et al., 2011). Our experiments show that TDP-43 promotes migration of lung cancer cells by binding to and regulating miR-423-3p. On the other hand, TDP-43 may also play a role in suppressing cancer development. More than 20 miRNAs were reported to be prognostic markers of non-small cell lung cancer (NSCLC) (Fanini et al., 2011; Skrzypski et al., 2011). Survival analyses indicate that TDP-43-regulated miRNAs are correlated with the survival of cancer patients and that they could potentially be used as cancer prognostic markers. In the case of miR-500a-3p, TDP-43 binds to the mature miR-500a-3p sequence, and miR-500a-3p expression is significantly down-regulated by TDP-43 knockdown. Though miR-500a-3p was not differentially expressed in LUSC samples versus control, it did have a significant positive correlation with TDP-43. In contrast to the possible role of TDP-43 in promoting lung cancer by regulating miR-423-3p, patients with low expression level of miR-500a-3p have poor prognosis, suggesting that TDP-43 may have a suppressive role in cancer by regulating miR-500a-3p.

Our data reveal *LIF* and *PAPPA* as potential targets of miR-500a-3p in lung cancer cells. *LIF* has been identified as a metastatic factor in rhabdomyosarcomas (Wysoczynski et al., 2007). A new lung cancer susceptibility locus located downstream of *LIF* were found through genome-wide association studies in Han Chinese (Hu et al., 2011). *PAPPA* has been shown to promote lung cancer growth and progression (Pan et al., 2012). Though miR-500a-3p overexpression and the luciferase assay showed miR-500a-3p affecting *LIF* expression, *PAPPA* was only affected after miR-500a-3p overexpression; the possibility that miR-500a-3p regulates PAPPA by interacting with binding sites outside its 3′UTR remains to be resolved by future studies. Nevertheless, miR-500a-3p may serve as a tumor suppressor through inhibiting *LIF* and *PAPPA*, and TDP-43 might contribute to inhibiting cancer progression by modulating miR-500a-3p target genes.

Taken together, these findings provide new insights into the involvement of TDP-43 in miRNA biogenesis, and suggest complex roles of TDP-43 in carcinogenesis. Our data suggest a working model for TDP-43, in which TDP-43 regulates miRNA expression and function in five ways. First, TDP-43 could interact with (1) Drosha and/or (2) Dicer to affect miRNA biogenesis and (3) regulate isomiR patterns. Then, (4) TDP-43 could regulate miRNA arm selection through mechanisms that remain to be elucidated. Lastly, (5) TDP-43 could bind to the mature miRNAs. MiRNA expression changes and binding of TDP-43 to miRNAs may affect their functional activities. Dysregulation of these processes may contribute to pathogenesis of various diseases. Our work suggests the complexity and multifaceted roles of TDP-43 in regulating microRNA processing and function. Clearly, further studies are necessary to elucidate mechanisms by which TDP-43 act in the pathogenesis of various human diseases, including cancer.

## Materials and Methods

### Reagents, cell cultures, and transfection

The polyclonal anti-TDP-43 antibody (Abcam) and monoclonal anti-beta-tubulin (BD Pharmingen) were obtained from corresponding vendors.

Human neuroblastoma cells (SH-SY-5Y), human glioma cells (SNB-19), human non-small cell lung cancer cells (H1299), and mouse HT22 cells were cultured in DMEM supplemented with 10% fetal bovine serum at 5% CO_2_ and 37°C. Human and mouse TDP-43 siGENOME SMARTpool oligos were purchased from Dharmacon. The siRNA oligos were transfected into the cells by lipofectamine2000 (Invitrogen) for 48 h followed by a second transfection for another 72 h. SiGENOME Non-Targeting siRNA Pool #2 oligos (Dharmacon) were used as a negative control. Mimetics and inhibitors of corresponding miRNAs were purchased from Genepharma company, and transfected into cells using lipofectamine2000 for 48 h.

### Western blotting

Whole cell lysates from non-targeting siRNAs or TDP-43 siRNAs transfected cells were obtained using lysis buffer (50 mmol/L Tris-HCl (pH 6.8), 10% glycerol, 2.5% β-mercaptoethanol, 2% SDS, 0.1% Bromophenol Blue). Cell lysate was loaded onto 10% SDS-PAGE and transferred onto Polyvinylidene Fluoride membranes. Membranes were incubated with rabbit anti-TDP-43 antibody (1:1000; Abcam). Mouse anti-β-tubulin (1:2500; BD Pharmingen) was used as an internal control.

### Total RNA extraction and small RNA sequencing

Following two rounds of transfection with the control or TDP-43 siRNAs, cells were lysed in Trizol (Invitrogen) for total RNA extraction with DNA removed by treating samples using RNase-free DNase I (Roche). The RNA yield was determined by UV absorbance spectroscopy (GE) and RNA quality was checked using 1% formaldehyde-agarose gel electrophoresis. Small RNA libraries (16–52 nt) were constructed using the TruSeq Small RNA Sample Prep Kit (Illumina) and sequenced using an Illumina Genome Analyzer IIx platform (one sample per lane), with one sample being sequenced from each condition (TDP-43 siRNA or control) for each cell line.

### Quantitative RT-PCR (qRT-PCR) analyses

Quantitative RT-PCR was performed using the Rotor-Q qRT-PCR instrument (Qiagen). Total RNA treated with the Turbo DNA-free™ Kit (Ambion) was used. The levels of miRNAs were quantified using the NCode™ VILO™ miRNA qRT-PCR kit (Invitrogen) and normalized with the U6 small nuclear RNA (U6 snRNA). The expression of corresponding miRNA target genes was measured using TransScript II Green One-Step qRT-PCR SuperMix (Transgen) with beta-actin and GAPDH as internal controls. All reactions, including reverse transcriptions and PCRs, were run in triplicates in at least 3 independent experiments. Primers used are listed in Table S15.

### RNA immunoprecipitation (RIP) assay

The RIP experiment protocol was described in our previously published work (Fan et al., 2014). Briefly, 2 × 10^7^ SH-SY5Y cells were collected, resuspended in 2 mL RIP lysis buffer [50 mmol/L Tris-HCl (pH 7.4), 1 mmol/L EDTA (pH 8.0), 250 mmol/L NaCl, 0.5% NP-40, 1 mmol/L PMSF, 1× Phosphatase Inhibitor Cocktail (sigma), 1× Protease Inhibitor Cocktail (sigma), and 0.1 U/mL of RNasin (Promega)] on ice for 15 min, and centrifuged at 4°C, 13,300 rpm for 30 min (Eppendorf). An antibody against TDP-43 (Abcam, IP grade) or rabbit IgG control (Millipore) was added to the lysate with gentle rotation at 4°C for 1 h. Protein A beads were added and incubated for an additional 3 h at 4°C with gentle rotation. Beads were collected at 4°C and centrifuged at 2,000 rpm for 2 min. They were then washed for three times in RIP buffer and resuspended in 1 mL Trizol. RNA was subsequently isolated for qRT-PCR analysis.

### Electrophoretic mobility shift assay (EMSA)

Biotinylated miRNAs were purchased from Takara. The coding region of TDP-43 was amplified with the following primers: TDP-FATGTCTGAATATATTCGGGT, TDP-R CTACATTCCCCAGCCAGAAG. The full-length TDP-43 tagged with His at the C-terminus (His-TDP-43 wild type) was constructed using the pEASY-E2 expression kit (Transgen).The wild-type His-TDP-43 was expressed in *Escherichia coli* stain Transetta (DE3) (Transgen) by incubation for 18 h at 16°C with 1 mmol/L IPTG. The resulting protein was then purified with Ni-NTA Fast Start Kit (Qiagen) in accordance with the manufacturer’s instructions. The biotin-labeled miRNAs were incubated with His-TDP-43 and the assays were carried out using LightShift Chemiluminescent RNA EMSA Kit (Thermo) following the manufacturer’s instruction.

### RNA pull-down assay

Cells were lysed with RIP lysis buffer (same as above) and then centrifuged. The lysate was split into four parts: one 100 μL aliquot for input, and three 300 μL aliquots for negative control, sense strand and antisense strand RNA, respectively. Biotin-labeled RNA was added to the lysate and incubated at 4°C for 3 h with gentle agitation. Streptavidin-coated magnetic beads (Invitrogen) were blocked for 2 h at 4°C in lysis buffer containing 1 mg/mL yeast tRNA (Ambion) and 1 mg/mL BSA (Ambion), and then washed twice with 1 mL lysis buffer. Then, the beads were suspended in the lysate for at least 3 h, and subsequently washed 5 times with 1 Ml wash buffer (10 mmol/L Tris (pH 8.0), 1 mmol/L EDTA, 0.5 mol/L NaCl). The beads were then boiled for 5 min in 0.1% SDS for dissociation and then subjected to Western blotting as described above.

### Transwell migration assay

For the *in vitro* cell migration assay, 5 × 10^4^ cells were suspended in 0.5 mL DMEM without serum, and then plated into the transwell inserts (BD Biosciences). To the bottom well, 0.75 mL DMEM with serum was added. Cells were incubated for 12 h, fixed in 75% ethanol for 10 min, and stained by crystal violet for 30 min. Cells that migrated cross the membrane were counted under a microscope from 6 randomly selected fields (at 200× magnification).

### Dual luciferase reporter assay

The 3′UTR sequences of LIF or PAPPA were inserted into the p-sicheck2 (Promega) vector. Mutants of psicheck2-LIF-3′-UTR or psicheck2-PAPPA-3′-UTR were obtained using the Fast Mutagenesis System (Transgen). H1299 cells were cotransfected with the 3′UTR reporter constructs and either control or miR-500a mimics in 24-well plates. After 24 h, luciferase activity was measured using the dual luciferase reporter assay system (Promega) according to the manufacturer’s instructions.

### Small RNA sequencing data analyses

#### Trimming of adapters and mapping to genomes

First the 3′ end adapters of reads generated by Illumina Genome Analyzer IIx were trimmed using The Flexible Barcode and Adapter Remover (FLEXBAR, Dodt et al., 2012). The minimum overlap between adapter and read was set to 6 bases. Two mismatches were allowed. Reads with length less than 10 bases after trimming adapters were removed from the data. If the adapter sequence was not found at the 3′ end, the last 6 bases were trimmed. The trimmed reads were then grouped into unique reads. Unique reads with read counts of 3 or more were mapped to either the human (UCSC hg19) or mouse (UCSC mm9) genomes using bowtie (Langmead et al., 2009). Reads mapping to the respective genomes without mismatches were then used to obtain expression profiles for the miRNAs. When obtaining expression levels of isomiRs with non-template additions at 3′ end, three mismatches were allowed.

#### Sequences of non-coding RNAs

The sequences of annotated pre-miRNAs and mature miRNAs were obtained from miRBase Release 18 (Kozomara and Griffiths-Jones, 2011). Ribosomal RNA sequences were extracted from the Silva database (http://www.arb-silva.de/; Quast et al., 2012), Ensembl (GRCh37 and NCBIM37) and Functional RNA database (fRNAdb) (archived at http://togodb.biosciencedbc.jp/togodb/view/frnadb_summary#en; Mituyama et al., 2009); sequences of tRNA from the Genomic tRNA Database (http://gtrnadb.ucsc.edu/; Chan and Lowe, 2009); snRNA sequences from Ensembl (GRCh37 and NCBIM37) and the fRNAdb; human snoRNA sequences from the snoRNABase (https://www-snorna.biotoul.fr; Lestrade and Weber, 2006) and Ensembl GRCh37; mouse snoRNA sequences from Ensembl NCBIM37 and the fRNAdb; piRNA sequences from the fRNAdb; and repeated elements from the Repbase (http://www.girinst.org/repbase/; Bao et al., 2015). We obtained the coordinates of exons and introns from UCSC Table Browser (http://genome.ucsc.edu), and subsequently extracted the sequences from the genomes using a Python script.

#### miRNA and isomiR expression profiling

Genomic coordinates of mature miRNAs were extracted (in BED format) from the annotation file in miRBase version 18. Genomic coordinates of reads mapping to the genome without mismatch were obtained (in BED format) using a Python script from mapping results. Only reads with length from 17 to 26 bases were selected. BEDTools (Version 2.9.0) (Quinlan and Hall, 2010) was used to compare the genomic coordinates to find reads overlapping with mature miRNAs. We considered reads overlapping with mature miRNAs if at least 17 bases are included in miRNAs and their isomiRs. Apart from miRNAs matching the miRBase sequences, we also counted reads mapping to each type of isomiR. To obtain the expression of isomiRs with non-template extension at 3′ end, we first mapped reads to the respective miRNAs sequences with one mismatch, and then counted reads that were one nucleotide longer than the miRBase sequence but else matched the miRBases sequence perfectly.

### Differential expression analysis

The number of reads mapping to miRNAs and their isomiRs were used to estimate their expression levels in the 3 pairs of libraries (SNB-19_siCtrl VS SNB-19_siTDP, SY-5Y_siCtrl VS SY-5Y_siTDP and HT22_siCtrl VS HT22_siTDP). We applied a Bayesian method developed by Audic and Claverie (Audic and Claverie, 1997) to identify differentially expressed miRNAs (*P*-value < 0.001). The fold change in miRNA expression between the 3 pairs of libraries was also calculated. The Chi-squared test was employed to calculate the statistical significance of changes in isomiR expression patterns after TDP-43 knockdown. We applied filter criteria to select miRNAs with isomiR pattern changes with high confidence: (1) total expression of at least 1000 counts in at least one condition; (2) the ratio of at least one isomiR variant to the total reads needed to change by at least 5% between conditions. Finally, we calculated the ratio of reads mapping to the 5p arm versus the 3p arm, and we selected miRNAs with significantly changing ratios if the ratio changed by 1.5-fold in either direction.

### Prediction of novel miRNA candidates

From the collection of reads mapping to the genome without mismatches we removed reads mapping to pre-miRNAs, tRNAs, rRNAs, snoRNAs, snRNAs, and piRNAs. The remaining reads were used to predict novel miRNA candidates using the updated miRanalyzer webserver (Hackenberg et al., 2011). miRanalyzer integrates at least 8 features, such as the secondary structure, to train five random forest models for the prediction of novel miRNA candidates.

### Functional annotation of differentially expressed TDP-43-regulated miRNAs

#### miR2Disease annotation and literature search

For each miRNA predicted to be regulated by TDP-43, miRNA associated diseases were collected from the miR2Disease database (Jiang et al., 2009). These references were compiled with our own literature search for these miRNAs.

#### Data collection for functional annotation analysis pipeline

See Figure S2 for a summary of the analysis pipeline. From The Cancer Genome Atlas database for lung squamous cell carcinoma and lung adenocarcinoma samples (Collins and Barker, 2007), we extracted the data from all samples that had paired miRNA-Seq and RNA-SeqV2 profiles available as of July 2014 (330 tumor, 37 control for LUSC; 422 tumor, 19 control for LUAD). From the precalculated human target predictions from miRanda (Betel et al., 2010; http://www.microrna.org/microrna/getDownloads.do), a matrix was generated using a python script reporting the number of binding sites for each miRNA-mRNA interaction in humans. Only predicted sites with a “good mirSVR” score were used, irrespective of conservation. A perl script was then used to assign the TCGA raw miRNA counts (*.isoform.quantification.txt files) to the mature miRNAs, as defined by miRBase version 21 (Kozomara and Griffiths-Jones, 2014). Another perl script was used to isolate the mRNA expression estimates (*.isoforms.normalized_results files) for the next steps.

#### ProMISe analysis

ProMISe is a recently developed technique (Li et al., 2014) that incorporates information about the number of binding sites a miRNA has on a target gene as well as expression levels of both the miRNAs and the target genes. Unique to ProMISe, though, is the generation of a competition model of miRNAs competing for a particular mRNA, and mRNAs competing to be inhibited by a particular miRNA. The joint model of these two competition models outperforms all other available miRNA-mRNA interaction prediction tools, and has the additional advantage of predicting these interactions within a single sample. For our data, the matrix from the miRanda predictions, the processed miRNA expression profiles, and the normalized mRNA isoform expression profiles were used as input for ProMISe, using the “joint model”, to generate for each sample a “ProMISe signal” consisting of a probability matrix of any particular miRNA targeting any particular gene. From the ProMISe signature for each sample, all miRNA-mRNA interactions with non-zero probability were counted as predicted miRNAs targets for that sample. For each miRNA, only interactions seen in all samples were included as a “predicted target” for downstream analyses.

#### Differential expression analysis and ranking transcripts

The isoform counts for miRNAs and mRNAs were submitted to DESeq2 (Love et al., 2014) for differential expression analysis using the standard settings. For miRNAs, the raw aggregated counts for mature miRNAs were used. For mRNAs, the RSEM normalized estimated counts were used; this is analogous to using salmon-derived estimated transcript abundances, as described in a recent paper (Soneson et al., 2016). In order to rank the transcripts for the Fatiscan step, an “adjusted rank” was used to give the most weight to transcripts that had the most expression, the most log-fold change, and the most statistically significant change. If the transcript had a base mean of 30 or less, then its rank was log10 of its base mean plus the absolute value of its log2 fold change; otherwise, its rank was those two items plus the absolute value of log10 of its adjusted *P*-value. Then the rank was given the same sign as the transcripts’ fold change (negative for down-regulated; positive for up-regulated).

#### Fatiscan analysis

Fatiscan (Al-Shahrour et al., 2007a) is a tool that is threshold-independent, using a heuristic to define a partition of a ranked list of genes or transcripts to identify whether a set of them are overrepresented among the most up-regulated or most down-regulated. In our case, we submitted a list of custom annotations based on the ProMISe results, with each transcript annotated with the miRNAs that target them, as well as the “adjusted rank” list generated in the previous step. We then ran Fatiscan with the options “remove duplicates”, “Fatiscan” model, “Two-tailed Fisher’s Exact Test”, and our custom miRNA annotations as the database to test. The results were downloaded and the adjusted *P*-values were extracted.

#### Selecting candidate miRNAs

From there, reminiscent of the technique used in SPIA (Tarca et al., 2009) of combining two dimensions of data, we used their “normal inversion” method to combine the DESeq2 adjusted *P*-value and the Fatiscan adjusted *P*-value for each miRNA. This combined *P*-value was then adjusted using the Benjamini-Hochberg method. We identified all miRNAs that had an adjusted combined *P*-valued < 0.05, and then applied three criteria to select candidate miRNAs: (A) the miRNA had to have a DESeq2 differential expression FDR < 0.1; (B) the targets of the miRNA had to be changing in the opposite direction (if the miRNA is up-regulated, the targets must be down-regulated, and vice versa); (C) the miRNA expression profile had to have a statistically significant correlation with the TDP-43 expression profile (FDR < 0.1). To calculate the correlation, we extracted out the TDP-43 normalized gene counts from the TCGA data, and then performed a Pearson correlation of the TDP-43 gene counts against each miRNA’s normalized counts (as calculated by DESeq2). We took the *P*-value of that correlation, and adjusted it using the Benjamini-Hochberg method. The resulting list of miRNAs for each combination of miRNA-mRNA interactions (down-regulated miRNAs targeting up-regulated mRNAs, and vice versa for LUAD and LUSC each) were submitted for the FatiGO step.

#### FatiGO analysis

To generate a list of functional annotations, the transcripts identified extracted from the targets of each candidate miRNA. Four functional groups were tested separately: the down-regulated targets of up-regulated miRNAs and the up-regulated targets of down-regulated miRNAs for LUAD and LUSC each. The first step was to convert the UCSC IDs to gene names. A Perl script with the June 2011 TCGA human genome annotation (the annotation used at the time of data generation; available at https://www.synapse.org/#!Synapse:syn1356421), along with the current kgXref_table and the versions 5 and 6 from the UCSC database were used to construct a table converting the UCSC transcript IDs to gene names, with some manual updating of those names using the Ensembl and Unigene databases. The resulting lists were submitted as gene lists to FatiGO (Al-Shahrour et al., 2007b), as part of the Babelomics 4.3 suite (v4.babelomics.org, Medina et al., 2010). Each gene list was compared against the human genome; the gene ontology biological process, gene ontology molecular function, BIOCARTA, KEGG, and Reactome databases were tested using the default settings.

#### Construction of a predicted causal interaction network

From all of the above results, a network of predicted causal links, from TDP-43 to lung cancer through TDP-43-regulated miRNAs and their targets, was constructed based on the significant targets that had at least one annotation. The resulting interaction network graphic was constructed using the HiveR R package (http://academic.depauw.edu/~hanson/HiveR/HiveR.html), based on the principles of the Hive Plot (Krzywinski et al., 2012). A python script was used to convert the various attributes (e.g., rank) to hive plot characteristics (e.g. node color). Each item was treated as a node on one of three axes: miRNAs, mRNAs, and pathway terms. The miRNA-mRNA edges are significant interactions identified by our pipeline; the mRNA-term edges are significant annotations identified by the FatiGO step. The rank of the node was mapped to the radial distance; the signed log10 of the FDR was mapped to the color for the miRNA and mRNA nodes; the signed log10 of the Fatiscan result was mapped to the miRNA-mRNA edges; the database category was mapped to the pathway term node color and to the mRNA-term edges; finally, the number of connections was mapped to the size of each node.

### miRNA target genes prediction

Recomputed predictions from three methods were applied to predict miRNA target genes after survival analysis: miRanda (Enright et al., 2003), PITA (Kertesz et al., 2007) and TargetScan (Friedman et al., 2009). The cutoff of miRanda predictions total score was −14. The cutoff of PITA prediction score was −10. Predicted target genes of TargetScan without conserved target sites were removed.

### Patient survival analyses

Genes and miRNAs expression profiles of LUSC, LUAD, and GBM were obtained from The Cancer Genome Atlas (TCGA) (Hammerman et al., 2012; Verhaak et al., 2010). Cox regression (Cox, 1972) was applied to identify miRNAs and genes that are significantly associated with patient survival. The risk-score was defined as: risk-score = cox regression coefficient × expression level of miRNA/gene. Samples could be separated into two groups, high risk group and low risk group, by each miRNA/gene according to its risk-score. The log-rank test (Bland and Altman, 2004) was applied to test the survival differences between these two groups.

### Data availability

The small-RNA-Seq data is deposited in the Gene Expression Omnibus database with accession GSE85065. The analysis scripts for the small-RNA-Seq data and the survival analysis can be found on github: https://github.com/bighanchen/miRNA-seq. The code and steps needed to reproduce the functional annotation and predicted causal network pipeline can also be found on github: https://github.com/warrenmcg/TDP43_miRNA_Paper.

## Electronic supplementary material

Below is the link to the electronic supplementary material.

Supplementary material 1 (XLS 20 kb)

Supplementary material 2 (XLS 82 kb)

Supplementary material 3 (XLS 139 kb)

Supplementary material 4 (XLSX 16 kb)

Supplementary material 5 (XLS 77 kb)

Supplementary material 6 (XLS 71 kb)

Supplementary material 7 (XLS 58 kb)

Supplementary material 8 (XLSX 51 kb)

Supplementary material 9 (XLSX 170 kb)

Supplementary material 10 (XLSX 237 kb)

Supplementary material 11 (XLSX 615 kb)

Supplementary material 12 (XLSX 159 kb)

Supplementary material 13 (XLS 85 kb)

Supplementary material 14 (XLS 41 kb)

Supplementary material 15 (XLS 20 kb)

Supplementary material 16 (PDF 820 kb)

## References

[CR1] Abu-Elneel K, Liu T, Gazzaniga FS, Nishimura Y, Wall DP, Geschwind DH, Lao K, Kosik KS (2008). Heterogeneous dysregulation of microRNAs across the autism spectrum. Neurogenetics.

[CR2] Adams BD, Parsons C, Walker L, Zhang WC, Slack FJ (2017). Targeting noncoding RNAs in disease. J Clin Investig.

[CR4] Al-Shahrour F, Arbiza L, Dopazo H, Huerta-Cepas J, Mínguez P, Montaner D, Dopazo J (2007). From genes to functional classes in the study of biological systems. BMC Bioinform.

[CR5] Al-Shahrour F, Minguez P, Tarraga J, Medina I, Alloza E, Montaner D, Dopazo J (2007). FatiGO+: a functional profiling tool for genomic data. Integration of functional annotation, regulatory motifs and interaction data with microarray experiments. Nucleic Acids Res.

[CR6] Ambros V (2004). The functions of animal microRNAs. Nature.

[CR7] Ambros V (2011). MicroRNAs and developmental timing. Curr Opin Genet Dev.

[CR8] Audic S, Claverie JM (1997). The significance of digital gene expression profiles. Genome Res.

[CR9] Ayala YM, De Conti L, Avendano-Vazquez SE, Dhir A, Romano M, D’Ambrogio A, Tollervey J, Ule J, Baralle M, Buratti E (2011). TDP-43 regulates its mRNA levels through a negative feedback loop. EMBO J.

[CR10] Baccarini A, Chauhan H, Gardner Thomas J, Jayaprakash Anitha D, Sachidanandam R, Brown Brian D (2011). Kinetic analysis reveals the fate of a micrornA following target regulation in mammalian cells. Curr Biol.

[CR11] Baker RG, Hsu CJ, Lee D, Jordan MS, Maltzman JS, Hammer DA, Baumgart T, Koretzky GA (2009). The adapter protein SLP-76 mediates “outside-in” integrin signaling and function in T cells. Mol Cell Biol.

[CR13] Bao W, Kojima KK, Kohany O (2015). Repbase update, a database of repetitive elements in eukaryotic genomes. Mob DNA.

[CR14] Baralle M, Buratti E, Baralle FE (2013). The role of TDP-43 in the pathogenesis of ALS and FTLD. Biochem Soc Trans.

[CR15] Bartel DP (2009). MicroRNAs: target recognition and regulatory functions. Cell.

[CR17] Betel D, Koppal A, Agius P, Sander C, Leslie C (2010). Comprehensive modeling of microRNA targets predicts functional non-conserved and non-canonical sites. Genome Biol.

[CR18] Bland JM, Altman DG (2004). The logrank test. BMJ.

[CR19] Bracken CP, Scott HS, Goodall GJ (2016). A network-biology perspective of microRNA function and dysfunction in cancer. Nat Rev Genet..

[CR21] Buratti E, De Conti L, Stuani C, Romano M, Baralle M, Baralle F (2010). Nuclear factor TDP-43 can affect selected microRNA levels. FEBS J.

[CR22] Buratti E, Romano M, Baralle FE (2013). TDP-43 high throughput screening analyses in neurodegeneration: advantages and pitfalls. Mol Cell Neurosci.

[CR23] Calin GA, Croce CM (2006). MicroRNA signatures in human cancers. Nat Rev Cancer.

[CR25] Campos-Melo D, Droppelmann CA, Volkening K, Strong MJ (2014). RNA-binding proteins as molecular links between cancer and neurodegeneration. Biogerontology.

[CR26] Chan PP, Lowe TM (2009). GtRNAdb: a database of transfer RNA genes detected in genomic sequence. Nucleic Acids Res.

[CR27] Chen Y, Song Y, Wang Z, Yue Z, Xu H, Xing C, Liu Z (2010). Altered expression of MiR-148a and MiR-152 in gastrointestinal cancers and its clinical significance. J Gastrointest Surg.

[CR28] Collins FS, Barker AD (2007). Mapping the cancer genome. Sci Am.

[CR29] Cox DR (1972). Regression models and lift-tables. J R Stat Soc Ser B.

[CR30] Crawford M, Batte K, Yu L, Wu X, Nuovo GJ, Marsh CB, Otterson GA, Nana-Sinkam SP (2009). MicroRNA 133B targets pro-survival molecules MCL-1 and BCL2L2 in lung cancer. Biochem Biophys Res Commun.

[CR31] Cummins JM, He Y, Leary RJ, Pagliarini R, Diaz LA, Sjoblom T, Barad O, Bentwich Z, Szafranska AE, Labourier E (2006). The colorectal microRNAome. Proc Natl Acad Sci USA.

[CR32] Czech B, Hannon GJ (2011). Small RNA sorting: matchmaking for Argonautes. Nat Rev Genet.

[CR35] Delfino KR, Serao NV, Southey BR, Rodriguez-Zas SL (2011). Therapy-, gender- and race-specific microRNA markers, target genes and networks related to glioblastoma recurrence and survival. Cancer Genom Proteom.

[CR36] Dodt M, Roehr J, Ahmed R, Dieterich C (2012). FLEXBAR—flexible barcode and adapter processing for next-generation sequencing platforms. Biology.

[CR37] Enright AJ, John B, Gaul U, Tuschl T, Sander C, Marks DS (2003). MicroRNA targets in Drosophila. Genome Biol.

[CR38] Esquela-Kerscher A, Slack FJ (2006). Oncomirs—microRNAs with a role in cancer. Nat Rev Cancer.

[CR39] Fan Z, Chen X, Chen R (2014). Transcriptome-wide analysis of TDP-43 binding small RNAs identifies miR-NID1 (miR-8485), a novel miRNA that represses NRXN1 expression. Genomics.

[CR40] Fang HY, Chen SB, Guo DJ, Pan SY, Yu ZL (2011). Proteomic identification of differentially expressed proteins in curcumin-treated MCF-7 cells. Phytomedicine.

[CR41] Fanini F, Vannini I, Amadori D, Fabbri M (2011). Clinical implications of microRNAs in lung cancer. Semin Oncol.

[CR42] Fernandez-Valverde SL, Taft RJ, Mattick JS (2010). Dynamic isomiR regulation in Drosophila development. Rna.

[CR44] Freischmidt A, Müller K, Ludolph AC, Weishaupt JH (2013). Systemic dysregulation of TDP-43 binding microRNAs in amyotrophic lateral sclerosis. Acta Neuropathol Commun.

[CR45] Friedman RC, Farh KK, Burge CB, Bartel DP (2009). Most mammalian mRNAs are conserved targets of microRNAs. Genome Res.

[CR46] Gangnon KT, Maxwell ES (2011). Elecrophoretic mobility shift assay for characterizing RNA-protein interaction. Methods Mol Biol.

[CR47] Gascon E, Gao FB (2014). The emerging roles of microRNAs in the pathogenesis of frontotemporal dementia-amyotrophic lateral sclerosis (FTD-ALS) spectrum disorders. J Neurogenet.

[CR49] Gregory RI, Yan KP, Amuthan G, Chendrimada T, Doratotaj B, Cooch N, Shiekhattar R (2004). The Microprocessor complex mediates the genesis of microRNAs. Nature.

[CR50] Griffiths-Jones S, Hui JHL, Marco A, Ronshaugen M (2011). MicroRNA evolution by arm switching. EMBO Rep.

[CR52] Guo L, Yang Q, Lu J, Li H, Ge Q, Gu W, Bai Y, Lu Z (2011). A comprehensive survey of miRNA repertoire and 3’ addition events in the placentas of patients with pre-eclampsia from high-throughput sequencing. PLoS ONE.

[CR53] Hackenberg M, Rodriguez-Ezpeleta N, Aransay AM (2011). miRanalyzer: an update on the detection and analysis of microRNAs in high-throughput sequencing experiments. Nucleic Acids Res.

[CR54] Hammerman PS, Lawrence MS, Voet D, Jing R, Cibulskis K, Sivachenko A, Stojanov P, McKenna A, Lander ES, Gabriel S (2012). Comprehensive genomic characterization of squamous cell lung cancers. Nature.

[CR55] Hu Z, Wu C, Shi Y, Guo H, Zhao X, Yin Z, Yang L, Dai J, Hu L, Tan W (2011). A genome-wide association study identifies two new lung cancer susceptibility loci at 13q12.12 and 22q12.2 in Han Chinese. Nat Genet.

[CR56] Huang JF, Wang Y, Guo YJ, Sun SH (2010). Down-regulated microRNA-152 induces aberrant DNA methylation in Hepatitis B virus-related hepatocellular carcinoma by targeting DNA methyltransferase 1. Hepatology.

[CR57] Humphreys DT, Hynes CJ, Patel HR, Wei GH, Cannon L, Fatkin D, Suter CM, Clancy JL, Preiss T (2012). Complexity of murine cardiomyocyte miRNA biogenesis, sequence variant expression and function. PLoS One.

[CR58] Hurd PJ, Nelson CJ (2009). Advantages of next-generation sequencing versus the microarray in epigenetic research. Brief Funct Genom Proteom.

[CR59] Jiang Q, Wang Y, Hao Y, Juan L, Teng M, Zhang X, Li M, Wang G, Liu Y (2009). miR2Disease: a manually curated database for microRNA deregulation in human disease. Nucleic Acids Res.

[CR60] Jonas S, Izaurralde E (2015). Towards a molecular understanding of microRNA-mediated gene silencing. Nat Rev Genet..

[CR62] Kawahara Y, Mieda-Sato A (2012). TDP-43 promotes microRNA biogenesis as a component of the Drosha and Dicer complexes. Proc Natl Acad Sci USA.

[CR63] Kertesz M, Iovino N, Unnerstall U, Gaul U, Segal E (2007). The role of site accessibility in microRNA target recognition. Nat Genet.

[CR64] Khvorova A, Reynolds A, Jayasena SD (2003). Functional siRNAs and miRNAs exhibit strand bias. Cell.

[CR65] Kocerha J, Kouri N, Baker M, Finch N, DeJesus-Hernandez M, Gonzalez J, Chidamparam K, Josephs KA, Boeve BF, Graff-Radford NR (2011). Altered microRNA expression in frontotemporal lobar degeneration with TDP-43 pathologycaused by progranulin mutations. BMC Genome.

[CR66] Kong YW, Ferland-McCollough D, Jackson TJ, Bushell M (2012). microRNAs in cancer management. Lancet Oncol.

[CR67] Kozomara A, Griffiths-Jones S (2011). miRBase: integrating microRNA annotation and deep-sequencing data. Nucleic Acids Res.

[CR68] Kozomara A, Griffiths-Jones S (2014). miRBase: annotating high confidence microRNAs using deep sequencing data. Nucleic Acids Res.

[CR69] Krzywinski M, Birol I, Jones SJM, Marra MA (2012). Hive plots-rational approach to visualizing networks. Brief Bioinform.

[CR70] Kuo PH, Doudeva LG, Wang YT, Shen CKJ, Yuan HS (2009). Structural insights into TDP-43 in nucleic-acid binding and domain interactions. Nucleic Acids Res.

[CR71] Lagier-Tourenne C, Polymenidou M, Cleveland DW (2010). TDP-43 and FUS/TLS: emerging roles in RNA processing and neurodegeneration. Hum Mol Genet.

[CR72] Landgraf P, Rusu M, Sheridan R, Sewer A, Iovino N, Aravin A, Pfeffer S, Rice A, Kamphorst AO, Landthaler M (2007). A mammalian microRNA expression atlas based on small RNA library sequencing. Cell.

[CR73] Langmead B, Trapnell C, Pop M, Salzberg SL (2009). Ultrafast and memory-efficient alignment of short DNA sequences to the human genome. Genome Biol.

[CR75] Lee LW, Zhang S, Etheridge A, Ma L, Martin D, Galas D, Wang K (2010). Complexity of the microRNA repertoire revealed by next-generation sequencing. RNA.

[CR76] Lee EB, Lee VM, Trojanowski JQ (2012). Gains or losses: molecular mechanisms of TDP43-mediated neurodegeneration. Nat Rev Neurosci.

[CR77] Lestrade L, Weber MJ (2006). snoRNA-LBME-db, a comprehensive database of human H/ACA and C/D box snoRNAs. Nucleic Acids Res.

[CR78] Li S-C, Liao Y-L, Ho M-R, Tsai K-W, Lai C-H, Lin W-C (2012). miRNA arm selection and isomiR distribution in gastric cancer. BMC Genome.

[CR79] Li Z, Lu Y, Xu XL, Gao FB (2012). The FTD/ALS associated RNA binding protein TDP-43 regulates the robustness of neuronal specification through microRNA-9a in Drosophila. Hum Mol Genet.

[CR80] Li Y, Liang C, Wong K-C, Jin K, Zhang Z (2014). Inferring probabilistic miRNA–mRNA interaction signatures in cancers: a role-switch approach. Nucleic Acids Res.

[CR81] Lin J, Huang S, Wu S, Ding J, Zhao Y, Liang L, Tian Q, Zha R, Zhan R, He X (2011). MicroRNA-423 promotes cell growth and regulates G1/S transition by targeting p21Cip1/Waf1 in hepatocellular carcinoma. Carcinogenesis.

[CR82] Liu N, Abe M, Sabin Leah R, Hendriks G-J, Naqvi Ammar S, Yu Z, Cherry S, Bonini Nancy M (2011). The exoribonuclease nibbler controls 3′ end processing of micrornas in drosophila. Curr Biol.

[CR83] Love MI, Huber W, Anders S (2014). Moderated estimation of fold change and dispersion for RNA-seq data with DESeq2. Genome Biol.

[CR84] Luciano DJ (2004). RNA editing of a miRNA precursor. Rna.

[CR85] Ma L, Young J, Prabhala H, Pan E, Mestdagh P, Muth D, Teruya-Feldstein J, Reinhardt F, Onder TT, Valastyan S (2010). miR-9, a MYC/MYCN-activated microRNA, regulates E-cadherin and cancer metastasis. Nat Cell Biol.

[CR86] Maciotta S, Meregalli M, Torrente Y (2013). The involvement of microRNAs in neurodegenerative diseases. Front Cell Neurosci.

[CR87] Malone JH, Oliver B (2011). Microarrays, deep sequencing and the true measure of the transcriptome. BMC Biol.

[CR88] Marti E, Pantano L, Banez-Coronel M, Llorens F, Minones-Moyano E, Porta S, Sumoy L, Ferrer I, Estivill X (2010). A myriad of miRNA variants in control and Huntington’s disease brain regions detected by massively parallel sequencing. Nucleic Acids Res.

[CR89] Medina I, Carbonell J, Pulido L, Madeira SC, Goetz S, Conesa A, Tárraga J, Pascual-Montano A, Nogales-Cadenas R, Santoyo J (2010). Babelomics: an integrative platform for the analysis of transcriptomics, proteomics and genomic data with advanced functional profiling. Nucleic Acids Res.

[CR91] Mituyama T, Yamada K, Hattori E, Okida H, Ono Y, Terai G, Yoshizawa A, Komori T, Asai K (2009). The Functional RNA Database 3.0: databases to support mining and annotation of functional RNAs. Nucleic Acids Research.

[CR92] Moore MJ, Silver PA (2008). Global Analysis of mRNA splicing. RNA.

[CR93] Morin RD, O’Connor MD, Griffith M, Kuchenbauer F, Delaney A, Prabhu AL, Zhao Y, McDonald H, Zeng T, Hirst M (2008). Application of massively parallel sequencing to microRNA profiling and discovery in human embryonic stem cells. Genome Res.

[CR94] Mostovich LA, Prudnikova TY, Kondratov AG, Loginova D, Vavilov PV, Rykova VI, Sidorov SV, Pavlova TV, Kashuba VI, Zabarovsky ER (2011). Integrin alpha9 (ITGA9) expression and epigenetic silencing in human breast tumors. Cell Adhes Migr.

[CR95] Nelson PT, Wang W-X, Rajeev BW (2008). MicroRNAs (miRNAs) in neurodegenerative diseases. Brain Pathol.

[CR96] Noorbakhsh J, Lang AH, Mehta P (2013). Intrinsic noise of microRNA-regulated genes and the ceRNA hypothesis. PLoS ONE.

[CR97] Orang AV, Safaralizadeh R, Kazemzadeh-Bavili M (2014). Mechanisms of miRNA-mediated gene regulation from common downregulation to mRNA-specific upregulation. Int J Genom.

[CR98] Osella M, Bosia C, Corá D, Caselle M (2011). The role of incoherent MicroRNA-mediated feedforward loops in noise buffering. PLoS Comput Biol.

[CR99] Pan H, Hanada S, Zhao J, Mao L, Ma MZ (2012). Protein secretion is required for pregnancy-associated plasma protein-a to promote lung cancer growth in vivo. PLoS ONE.

[CR100] Park YY, Kim SB, Han HD, Sohn BH, Kim JH, Liang J, Lu Y, Rodriguez-Aguayo C, Lopez-Berestein G, Mills GB, Sood AK, Lee JS (2013). Tat-activating regulatory DNA-binding protein regulates glycolysis in hepatocellular carcinoma by regulating the platelet isoform of phosphofructokinase through microRNA 520. Hepatology.

[CR101] Parpart S, Wang XW (2013). MicroRNA regulation and its consequences in cancer. Curr Pathobiol Rep.

[CR102] Peritz T, Zeng F, Kannanayakal TJ, Kilk K, Eiríksdóttir E, Langel U, Eberwine J (2006). Immunoprecipitation of mRNA-protein complexes. Nat Protoc.

[CR103] Polymenidou M, Lagier-Tourenne C, Hutt KR, Huelga SC, Moran J, Liang TY, Ling SC, Sun E, Wancewicz E, Mazur C (2011). Long pre-mRNA depletion and RNA missplicing contribute to neuronal vulnerability from loss of TDP-43. Nat Neurosci.

[CR104] Postel-Vinay S, Véron AS, Tirode F, Pierron G, Reynaud S, Kovar H, Oberlin O, Lapouble E, Ballet S, Lucchesi C (2012). Common variants near TARDBP and EGR2 are associated with susceptibility to Ewing sarcoma. Nat Genet.

[CR105] Quast C, Pruesse E, Yilmaz P, Gerken J, Schweer T, Yarza P, Peplies J, Glockner FO (2012). The SILVA ribosomal RNA gene database project: improved data processing and web-based tools. Nucleic Acids Res.

[CR106] Quinlan AR, Hall IM (2010). BEDTools: a flexible suite of utilities for comparing genomic features. Bioinformatics.

[CR107] Ratti A, Buratti E (2016). Physiological functions and pathobiology of TDP-43 and FUS/TLS proteins. J Neurochem.

[CR109] Ruby JG, Jan C, Player C, Axtell MJ, Lee W, Nusbaum C, Ge H, Bartel DP (2006). Large-scale sequencing reveals 21U-RNAs and additional microRNAs and endogenous siRNAs in C. elegans. Cell.

[CR110] Schwarz DS, Hutvagner G, Du T, Xu Z, Aronin N, Zamore PD (2003). Asymmetry in the assembly of the RNAi enzyme complex. Cell.

[CR111] Sephton CF, Cenik C, Kucukural A, Dammer EB, Cenik B, Han Y, Dewey CM, Roth FP, Herz J, Peng J (2011). Identification of neuronal RNA targets of TDP-43-containingribonucleoprotein complexes. J Biol Chem.

[CR112] Skrzypski M, Dziadziuszko R, Jassem J (2011). MicroRNA in lung cancer diagnostics and treatment. Mutat Res.

[CR113] Soneson C, Love MI, Robinson MD (2016). Differential analyses for RNA-seq: transcript-level estimates improve gene-level inferences. F1000Research.

[CR115] Srinivasan S, Patric IR, Somasundaram K (2011). A ten-microRNA expression signature predicts survival in glioblastoma. PLoS One.

[CR116] Sriram G, Birge RB (2011). Emerging roles for crk in human cancer. Genes Cancer.

[CR117] Tan X, Qin W, Zhang L, Hang J, Li B, Zhang C, Wan J, Zhou F, Shao K, Sun Y (2011). A 5-microRNA signature for lung squamous cell carcinoma diagnosis and hsa-miR-31 for prognosis. Clin Cancer Res.

[CR118] Tarca AL, Drăghici S, Khatri P, Hassan SS, Mittal P, Kim J-S, Kim CJ, Kusanovic JP, Romero R (2009). A novel signaling pathway impact analysis. Bioinformatics.

[CR119] Teittinen KJ, Kärkkäinen P, Salonen J, Rönnholm G, Korkeamäki H, Vihinen M, Kalkkinen N, Lohi O (2012). Nucleolar proteins with altered expression in leukemic cell lines. Leukemia Res.

[CR120] Tollervey JR, Curk T, Rogelj B, Briese M, Cereda M, Kayikci M, Konig J, Hortobagyi T, Nishimura AL, Zupunski V (2011). Characterizing the RNA targets and position-dependent splicing regulation by TDP-43. Nat Neurosci.

[CR121] Tsuruta T, Kozaki K, Uesugi A, Furuta M, Hirasawa A, Imoto I, Susumu N, Aoki D, Inazawa J (2011). miR-152 is a tumor suppressor microRNA that is silenced by DNA hypermethylation in endometrial cancer. Cancer Res.

[CR122] Ule J, Jensen K, Mele A, Darnell RB (2005). CLIP: A method for identifying protein-RNA interaction sites in living cells. Methods.

[CR123] Verhaak RG, Hoadley KA, Purdom E, Wang V, Qi Y, Wilkerson MD, Miller CR, Ding L, Golub T, Mesirov JP (2010). Integrated genomic analysis identifies clinically relevant subtypes of glioblastoma characterized by abnormalities in PDGFRA, IDH1, EGFR, and NF1. Cancer Cell.

[CR124] Woo HH, Laszlo CF, Greco S, Chambers SK (2012). Regulation of colony stimulating factor-1 expression and ovarian cancer cell behavior in vitro by miR-128 and miR-152. Mol Cancer.

[CR125] Wysoczynski M, Miekus K, Jankowski K, Wanzeck J, Bertolone S, Janowska-Wieczorek A, Ratajczak J, Ratajczak MZ (2007). Leukemia inhibitory factor: a newly identified metastatic factor in rhabdomyosarcomas. Cancer Res.

[CR127] Xiao S, Sanelli T, Dib S, Sheps D, Findlater J, Bilbao J, Keith J, Zinman L, Rogaeva E, Robertson J (2011). RNA targets of TDP-43 identified by UV-CLIP are deregulated in ALS. Mol and Cell Neurosci.

[CR129] Yanaihara N, Caplen N, Bowman E, Seike M, Kumamoto K, Yi M, Stephens RM, Okamoto A, Yokota J, Tanaka T (2006). Unique microRNA molecular profiles in lung cancer diagnosis and prognosis. Cancer Cell.

[CR130] Yu J, Cao Q, Mehra R, Laxman B, Yu J, Tomlins SA, Creighton CJ, Dhanasekaran SM, Shen R, Chen G (2007). Integrative genomics analysis reveals silencing of β-adrenergic signaling by polycomb in prostate Cancer. Cancer Cell.

[CR131] Zhang Z, Almeida S, Lu Y, Nishimura AL, Peng L, Sun D, Wu B, Karydas AM, Tartaglia MC, Fong JC (2013). Downregulation of microRNA-9 in iPSC-derived neurons of FTD/ALS patients with TDP-43 mutations. PLoS One.

[CR132] Zhou X, Zhao F, Wang ZN, Song YX, Chang H, Chiang Y, Xu HM (2012). Altered expression of miR-152 and miR-148a in ovarian cancer is related to cell proliferation. Oncol Rep.

