# Supplementary material for: TDP-43 regulates cancer-associated microRNAs
Source: Protein Cell. 2017 Sep 26;9(10):848–66. doi: 10.1007/s13238-017-0480-9 (PMC6160384; doi:10.1007/s13238-017-0480-9)
Supplement: Supplementary file 16 — Supplementary material 16 (PDF 820 kb) [file 13238_2017_480_MOESM16_ESM.pdf]

SupplementaryFigurelegends

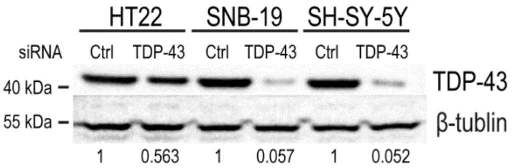

Supplementary Fig.1. Knockdown of TDP-43 in the three cell lines. β-tublinwas usedas loading control. Knockdown efficiencies: 44% in HT22, 94% in SNB-19, and 95% in SH-SY-5Y. Only the cells confirmed by western blot were used for high throughput sequencing.

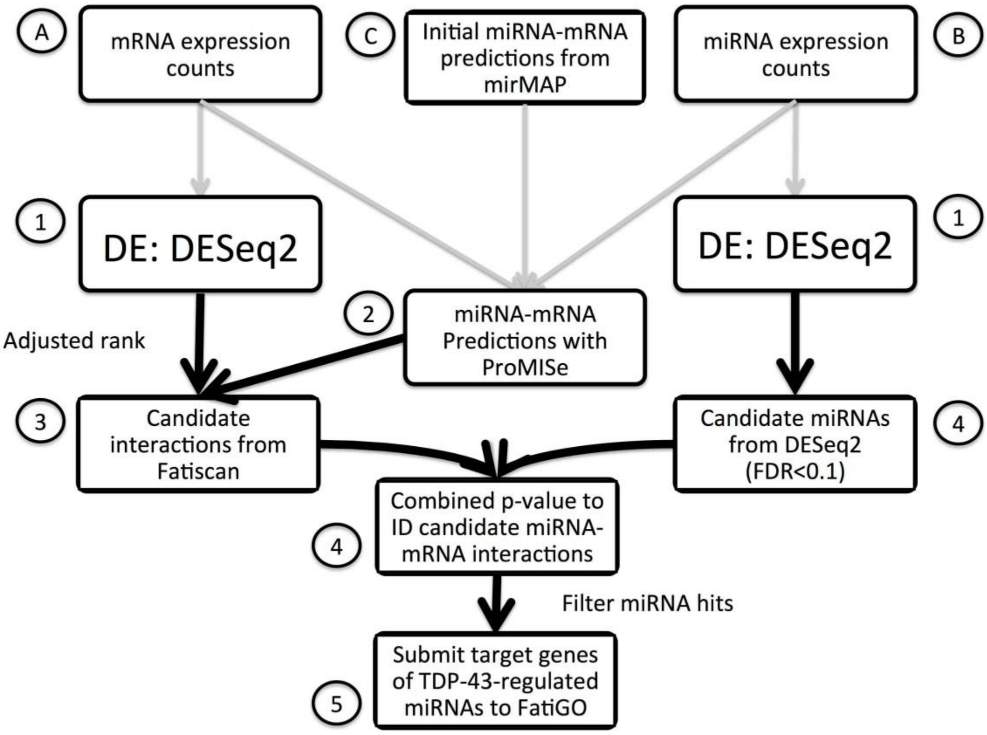

Supplementary Fig.2.A pipeline for functional annotation of differentially expressed TDP-43-regulated miRNAs.

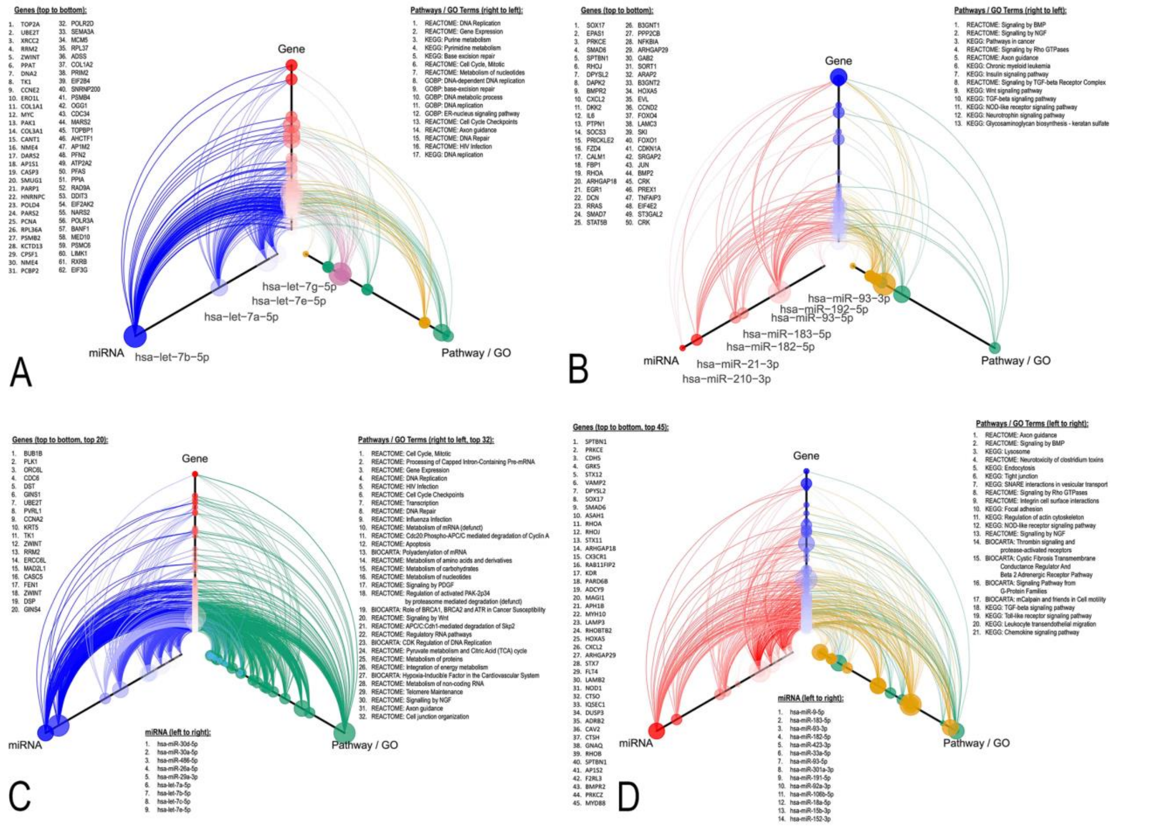

SupplementaryFig.3.The network graph of miRNA-mRNA interactions and the significant FatiGO terms associated with the target mRNAs in LUAD and LUSC samples. These are hive plots linking miRNAs to putative mRNA targets, and gene ontology terms identified to be significantly enriched in these diseases using Fatiscan. The radial distance is the rank of the nodes within their respective groups. The node size is proportional to the number of connections for a node. The color of the miRNA and gene nodes is related to the statistical significance of differential expression, and the color of the Pathway / GO term is related to the category. Finally, the color of the miRNA-mRNA connections is related to the statistical significance of the Fatiscan step, and the color of the mRNA-FatiGO connection is also related to the category. Note that some of the significant pathway terms are omitted for clarity. See supplementary tables 9 and 10 for the full list of significant pathways and the list of edges and nodes depicted. (A) down-regulated miRNAs targeting up-regulated transcripts in LUAD samples. (B) Up-regulated miRNAs targeting down-regulated transcripts in LUAD samples. (C) Down-regulated miRNAs targeting up-regulated transcripts in LUSC samples. (D) Up-regulated miRNAs targeting down-regulated transcripts in LUSC samples.

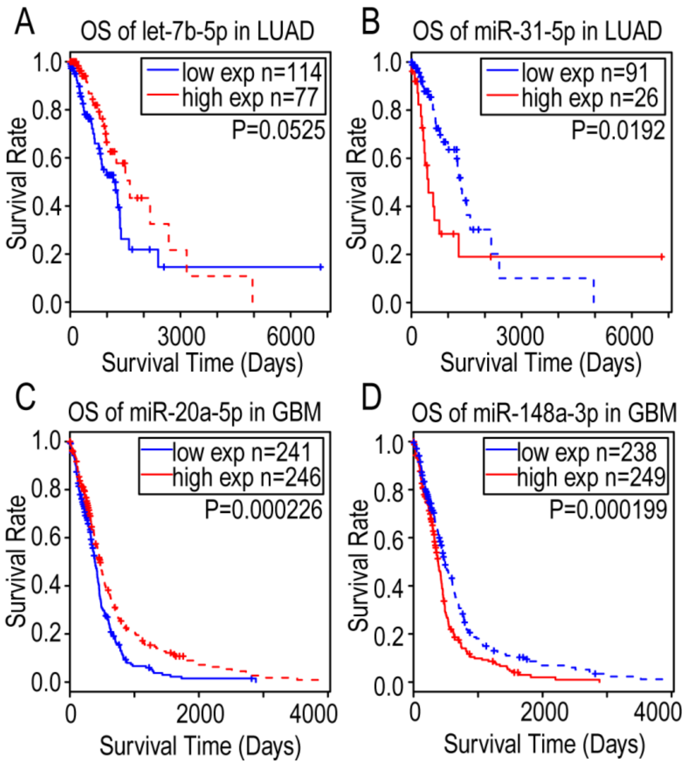

Supplementary Fig.4.Overall survival analysis of TDP-43 affected miRNAs in LUAD and GBM. Overall survival analysis for let-7b-5p (A) and miR-31-5p (B) in LUAD, and for miR-20a-5p (C) and miR-148a-3p (D) in GBM.p-values from log-rank test.

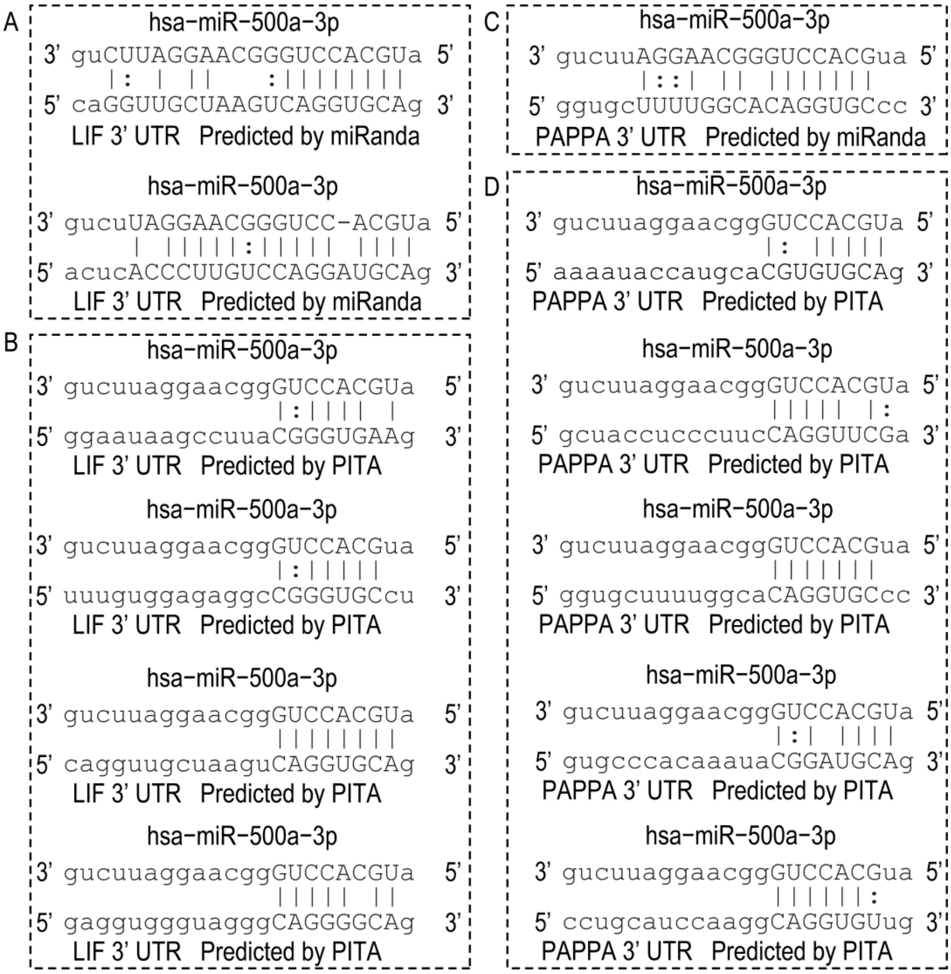

Supplementary Fig. 5. Predicted binding sites of miR-500a-3p in 3′UTR of LIF and PAPPA by miRanda and PITA. A. Binding sites in 3′UTR of LIF predicted by miRanda. B. Binding sites in 3′UTR of LIF predicted by PITA. C. Binding sites in 3′UTR of PAPPA predicted by miRanda. D. Binding sites in 3′UTR of PAPPA predicted by PITA.

SupplementaryTables

Supplementary Table1. Reads distribution of all libraries for RNA-seq.

Supplementary Table2.Differential expressed miRNAs in SH-SY-5Y, SNB-19 and HT22 cell lines after TDP-43 knockdown.

Supplementary Table3.IsomiR patterns between TDP-43 knockdown and control cell lines

Supplementary Table4.The unannotated arms and arm switching identified from high-throughput sequencing.

Supplementary Table5.5′ arm/3′ arm reads count ratio.

Supplementary Table6. Novel predicted miRNAs from high-throughput sequencing.

Supplementary Table7. TDP-43-regulated miRNAs associated diseases.The miRNAs in this table are both reported to be associated with cancer and up-/down-regulated in both SNB19 and SY5Y supported by RNA-seq and qRT-PCR, or changed in isomiR patterns or arm selections.

Supplementary Table8.miR2Disease annotation of TDP-43 regulated miRNAs.

Supplementary Table 9: a summary of the LUAD analysis. miRNAs that are significant from

the combined analysis are included, with a separate table for those that are differentially expressed and those that are not. There is a separate sheet to summarize the correlations for all miRNAs, and a final sheet to list the TDP-43-regulated miRNAs.

Supplementary Table 10: a summary of the LUSC analysis. miRNAs that are significant from the combined analysis are included, with a separate table for those that are differentially expressed and those that are not. There is a separate sheet to summarize the correlations for all miRNAs, and a final sheet to list the TDP-43-regulated miRNAs.

SupplementaryTable 11: a list of all of the cellular processes identified by FatiGO.

SupplementaryTable 12: a table to show the data to produce the hive plots shown in the Supplementary Figure 3).

Supplementary Table13. Prognostic miRNAs regulated by TDP-43.

Supplementary Table14. Survival analysis of miR-500a-3p target genes.

Supplementary Table15. Primers used in this study.
